# Problematizing the role of artificial intelligence in hiring and organizational inequalities: A multidisciplinary review

**DOI:** 10.1177/00187267251403902

**Published:** 2025-12-30

**Authors:** Karen D Hughes, Alla Konnikov, Nicole Denier, Yang Hu

**Affiliations:** University of Alberta, Canada, karen.hughes@ualberta.ca; Concordia University of Edmonton, Canada, alla.konnikov@concordia.ab.ca; Colby College, USA, nicole.denier@ualberta.ca; University of College London, UK, yang.hu@ucl.ac.uk

**Keywords:** inequality, new technology, human resource management, recruitment, personnel selection, employment law

## Abstract

What are the implications of the growing use of artificial intelligence (AI) in recruitment and hiring for organizational inequalities? While advocates suggest that AI is a groundbreaking tool that can enhance hiring precision, efficiency, diversity and fit, critics raise serious concerns around bias, fairness, and privacy. This review article critically advances this debate by drawing on diverse scholarship across computing and data sciences; human resource, management, and organization studies; social sciences; and law. Using a hybrid review approach that combines scoping and problematizing review methods, we examine the implications of algorithmic hiring for organizational inequalities. Our review identifies a multidisciplinary discussion marked by asymmetries in how key concerns are conceptualized; a clear and heightened potential for AI to conceal inequalities in hiring processes; and contestation over the regulation of algorithmic hiring. Building on Acker’s (2006) framework of ‘inequality regimes’, we propose the concept of *algorithmically-mediated inequality regimes* to highlight AI’s capacity for concealing and reproducing inequalities in hiring through enhanced *algorithmic invisibility* and the growing *legitimacy* of AI solutions. We propose an agenda for future research, policy, and practice, emphasizing the need for an interdisciplinary ‘chain of knowledge’ and a multi-stakeholder ‘chain of responsibility’ in AI application and regulation.

## Introduction

Hiring and recruitment practices are being dramatically reshaped by artificial intelligence (AI), sparking important debate over the implications for individual workers and organizational inequalities (Bornstein, 2018; Nawaz, 2019; Upadhyay and Khandelwal, 2018). Advocates view AI as an enticing ‘technosolution’ to perennial challenges in hiring, concerning accuracy, fit, efficiency and authenticity (Roemmich et al., 2023). Critics raise serious concerns about issues of bias, fairness and privacy (Aizenberg and Van Den Hoven, 2020; Burrell and Fourcade, 2021; Weiskopf and Hansen, 2023). While hiring processes have long relied on technologies – such as databases, resume screening software, and cybervetting (Berkelaar, 2017; Friedman and McCarthy, 2020) – it is clear that AI is ushering in a fundamentally new era. Today’s complex algorithmic ecosystems operate at unprecedented speed and scale, optimizing job postings, screening resumes, and analyzing candidates’ skills, body language, and speech to predict ‘ideal’ matches (Ajunwa and Green, 2019; Ajunwa, 2021b; Bornstein, 2018; Kelan, 2023; Manroop et al., 2024; Weiskopf and Hansen, 2023). Yet, while AI promises benefits – streamlining hiring processes for employers, and simplifying job searches for applicants – it also carries significant risks, potentially amplifying bias, and obscuring the mechanisms that sustain inequality.

Hiring is a critical and theoretically rich site for considering the implications of AI. It is widely acknowledged as a foundational gatekeeping mechanism within market-based capitalist societies that precedes and conditions all subsequent organizational processes (Acker, 2006; Kim, 2020). Unlike other human resource functions, impacting those already employed in organizations, hiring processes determine who is included and who is excluded, thus structuring economic opportunities and ‘life chances’ (Weber, 1978). Inequality produced in hiring processes has unique stakes, with the capacity to generate cascading cumulative effects on career trajectories, income, and social mobility (DiPrete and Eirich, 2006; Rivera, 2020). Individuals *not* hired cannot be evaluated or promoted and are thus excluded from the opportunities to perform and compete for rewards within organizations (Bills et al., 2017). As AI is rapidly integrated into hiring processes (SHRM, 2022), shaping access to rewards and opportunities, it is timely to examine whether and how AI is (re)shaping inequality.

Our work offers a multidisciplinary review of scholarship and debates currently grappling with the implications of AI for hiring inequalities. Ongoing debates are occurring across varied disciplines – including computing science (CS), human resource, management, and organization studies (HRMOS), the social sciences (SS), and law (LS) – highlighting that AI in hiring is an inherently multidisciplinary phenomenon. Yet, to the best of our knowledge, no review has explored this issue from a multidisciplinary perspective (e.g. Bankins et al., 2024; Basu et al., 2023; Burrell and Fourcade, 2021; Chen, 2023b; Kellogg et al., 2020). Embracing a multidisciplinary view is particularly important for understanding the role of AI in hiring, where technical, social, organizational, and legal considerations intersect to shape opportunities. As our review demonstrates, despite shared concerns, scholars often work in disciplinary silos, guided by distinct foci and assumptions, with limited exchange. This lack of cross-fertilization hinders a comprehensive understanding of how AI contributes to the (re)production of inequalities.

Theoretically, we employ Acker’s (2006) conceptualization of ‘inequality regimes’ to underscore the structural pervasiveness of organizational inequality and to problematize debates occurring across and within disciplines. Although Acker’s writing predates the rise of AI, her perspective is highly relevant to algorithmic hiring, where inequalities can be embedded within data, design, and the broader ecosystem. This approach underscores how multiple inequality-producing mechanisms can pervade routine organizational processes, remaining invisible while appearing legitimate and difficult to challenge.

Methodologically, we undertake a hybrid approach to reviewing and bringing this multidisciplinary scholarship together. First, we assess the breadth of the emerging debates using a scoping methodology (Arksey and O’Malley, 2005) to identify relevant scholarship in a rigorous manner. Specifically, we ask: *What key questions and concerns are being explored across disciplines regarding the role of AI in organizational hiring processes?* Second, we assess the depth of the emergent foci, ideas, and debates via a problematizing review (Alvesson and Sandberg, 2020) which questions underlying assumptions and frameworks in order to enable the development of new ideas and concepts. Specifically, we ask: *What underlying assumptions and approaches guide each discipline?* In answering these questions, we bring Acker’s framework of ‘inequality regimes’ (2006) into the digital era to problematize current debates and to conceptualize the place and the role of AI in (re)shaping and interacting with organizational inequality regimes, focusing on how its increasing pervasiveness may reinforce, reconfigure, or obscure inequalities.

Our multidisciplinary review makes several contributions to advancing knowledge about hiring and labour market inequalities in the digital age. First, we demonstrate that despite shared concerns about AI’s potential to exacerbate organizational inequality, there is a growing dominance of technical knowledge and perspective which risks marginalizing critical, alternative perspectives. Second, the current framing of AI as a solution to human bias narrows the focus to individual decision-makers and technical solutions, neglecting the pervasiveness of structural inequalities that are becoming further concealed within what we conceptualize as *algorithmically-mediated inequality regimes*. Third, we note that regulatory responses to AI are characterized by significant lag and contestation, with outcomes varying across global, national, regional, and local contexts. This complicates the development of coherent strategies to address AI’s far-reaching implications for hiring. It is only through a multidisciplinary lens that these patterns, tensions, and blind spots become visible. We situate our analysis within broad structures of power asymmetries, exacerbated by AI and digitization, recognizing that organizations do not operate in a vacuum. We conclude by outlining an agenda for future theorizing, research, and practice. We emphasize the need for interdisciplinary dialogue and multi-stakeholder ‘chain of responsibility’ to foster a more holistic understanding of AI’s transformative effects.

## Conceptual framework: Inequality regimes and AI

We extend Acker’s (2006) framework of inequality regimes to critically examine the implications of AI for hiring inequalities. ‘Inequality regimes’ are defined as ‘interrelated practices, processes, actions, and meanings that result in and maintain inequalities within organizations’ (443). Acker (2006) identifies hiring as a pivotal organizational process that structures access to opportunities and reinforces disparities across class, gender, and racial lines. AI is defined as computational systems that simulate human intelligence to perform tasks such as learning, reasoning, and decision-making (Russell and Norvig, 2021). In hiring, AI encompasses technologies including natural language processing, machine learning, and facial recognition, among others, which are used to optimize job postings, analyze resumes, and assess candidates’ speech and behaviour.

The use of AI in hiring both echoes and extends Acker’s theorization of inequality regimes. Acker (2006) argues that inequality-producing mechanisms often operate invisibly within routine processes, seemingly legitimate and difficult to challenge. It is only when they become visible that their legitimacy is questioned. Yet, AI solutions often operate in the opposite direction – first, with almost ubiquitous legitimacy, as evidenced by the growing normalization of digital trace collection and usage (Elliott, 2019; Zuboff, 2019), and second, with heightened invisibility, where AI functions as a ‘black box’ that renders decision-making processes more opaque and difficult to trace (Ajunwa, 2020b; Pasquale, 2015). These changes are situated within broader systemic changes conceptualized by Zuboff (2019) as *surveillance capitalism* – a new economic logic in which digital tracing and data extraction serve to generate profit and intensify power asymmetries between corporations and individuals. As Pasquale (2015) and Zuboff (2019) argue, these effects are not mere byproducts of the technology but are intentional, thus reproducing and magnifying power asymmetries.

Hiring offers an ultimate example of how these power dynamics operate (Ajunwa and Green, 2019). Despite being a two-sided process, power relations within hiring are highly asymmetric. Employers make high-stakes decisions defining who will be granted access to a job opportunity, a key life-chance structuring experience within market-based, capitalist, economies. While hiring is driven by the search for the ‘ideal worker’, the stated criteria may not be neutral but gendered, racialized and intersectional, and shaped by understandings of class and social location (Acker, 2006). Despite efforts to the contrary, hiring procedures and outcomes often remain enigmatic, leaving job seekers struggling to adapt their profiles and maximize their odds of being seen as an ‘ideal candidate’ (Ajunwa and Green, 2019). The increasing automation and augmentation of hiring processes exacerbate these complexities, posing new concerns (Kellogg et al., 2020; Newman et al., 2020). Amongst these are the growing legitimacy of complex algorithmic hiring tools, with magnified, often unregulated access to data (Zuboff, 2019); the increased opaqueness of decision-making (Pasquale, 2015); and growing power asymmetries in hiring (Ajunwa, 2020a). This constrains the agency of job seekers, limiting their choices and ability to understand or shape decisions made about them.

Despite this fundamental transformation of hiring, the role of AI in (re)shaping hiring inequality has not yet been thoroughly examined. Important reviews of organizational inequality, such as Amis et al. (2020), discuss hiring’s central role in producing inequality but omit considerations of AI. Our multidisciplinary review aims to clarify the relationship between AI, hiring, and inequality. Extending Acker’s framework to the digital era helps to problematize the way technological developments intertwine with inequality regimes.

## Methodology

Our positionality grounds how we engage with this review. As a team of four social scientists with distinct backgrounds, and shared expertise in social, organizational, and labour market inequality, we bring three vantage points to this review. First, we view social inequality as a structural rather than an individual phenomenon. Accordingly, work and organizations are structures in which inequality is produced and reproduced. Second, inspired by constructivist perspectives (Alvesson and Sköldberg, 2018), we view knowledge as socially constructed, not neutral, and shaped by power dynamics. Third, through shared experiences of conducting research on the implications of AI for labour market inequality as a part of a multidisciplinary team (including computing, statistics, and social science scholars), we are practically attuned to how different fields study this issue. This informs our attention to how knowledge is produced, how certain ideas gain traction while others are underexplored, and how disciplinary assumptions, including our own, shape knowledge.

Similarly, we recognize that our interpretive choices are shaped by our epistemological stances and disciplinary assumptions. For instance, perceiving social and organizational inequality as a structural phenomenon makes us more attentive to whether and how inequality is defined and discussed in different bodies of scholarship, and to whether these discussions occur at structural or individual levels. Our approach to knowledge formation also made us attentive to the language and terminology used to discuss inequality, and to how language reflects underlying disciplinary assumptions (e.g. ‘bias’ and ‘debiasing’ signalling a technosolutionist approach). Our experience of working in multidisciplinary teams has taught us to look within and across disciplines, building deep intradisciplinary knowledge and creating ‘bridges’ through interdisciplinary perspectives.

**Figure 1.**
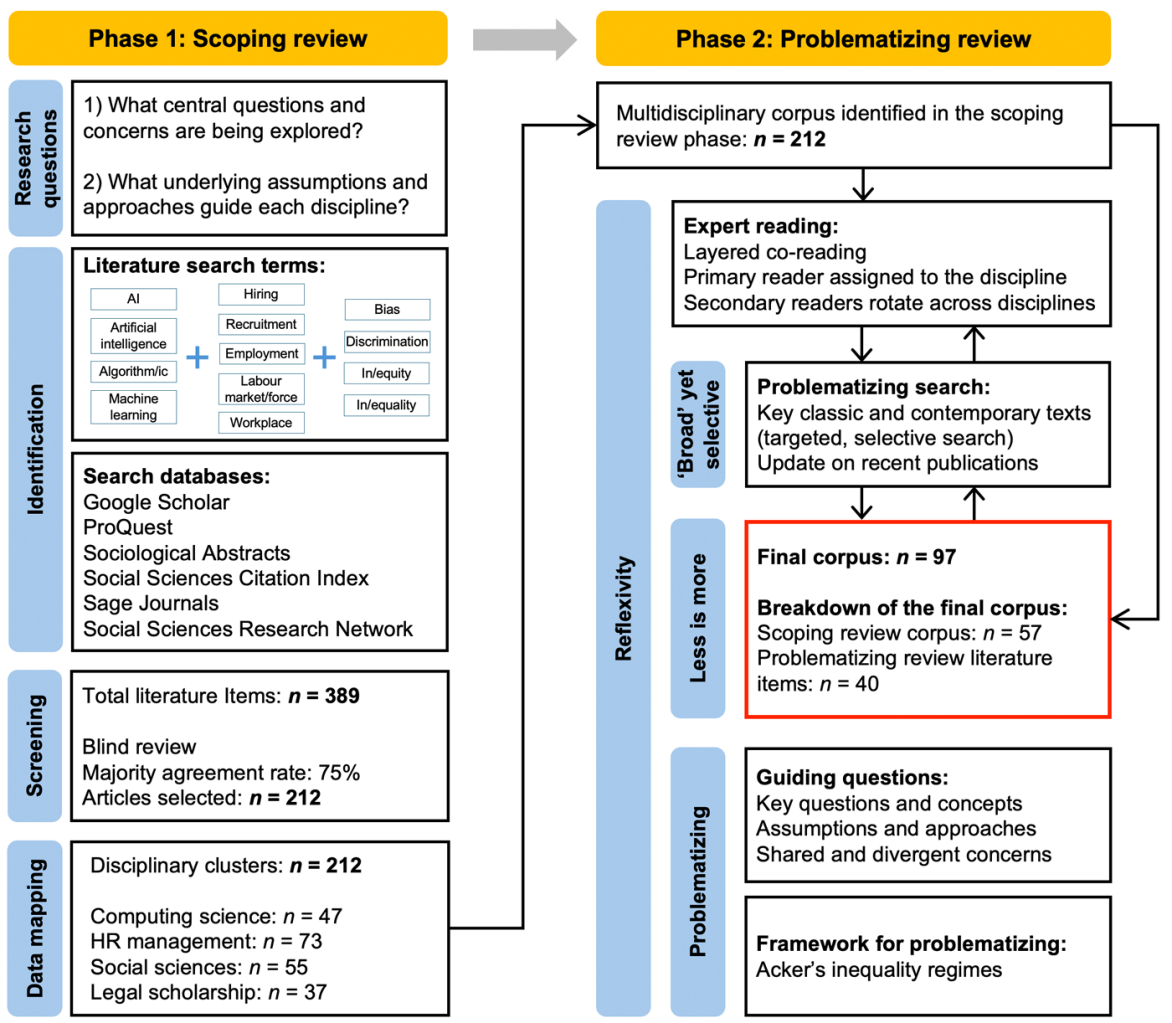
Map of the hybrid review approach.

Our hybrid methodology, combining scoping and problematizing methods, highlights our explorative stance, focusing on *what* knowledge is being created (Arksey and O’Malley, 2005), and our constructivist stance, focusing on *how* knowledge is being created (Alvesson and Sköldberg, 2018). Ultimately, our aim is not simply to map key debates and participating disciplines, but to critically question how knowledge is being shaped (see Supplemental Material C for further details). This hybrid methodology is essential for addressing the complexity of AI and hiring research as it evolves rapidly across disciplines. A key strength of a scoping review is its capacity to assess the breadth of a growing body of work that defines the ‘literature’ and to map emerging ideas and questions. The problematizing review complements and deepens this by critically engaging with key debates and assumptions. Together, this hybrid approach (see Figure 1) supports our aim of fostering interdisciplinary dialogue on AI’s role in hiring and its implications for organizational and social inequality.

### Phase 1: Scoping review

A scoping methodology is ideally suited for mapping emerging, complex, and evolving fields (Mays et al., 2001: 194). It provides flexibility to incorporate work from distinct disciplines and traditions (Daudt et al., 2013), rooted in an explorative, question-driven approach to knowledge creation, rather than a hypothesis-oriented one. While a scoping review methodology does not incorporate explicit quality assessment – a key distinction from the systematic review – it is nonetheless a rigorous, multi-step process aimed at maximizing breadth and inclusion of non-mainstream scholarship (Arksey and O’Malley, 2005). The steps are presented on the left-hand side of Figure 1.

In the first step, we worked to identify relevant literature. Guided by our research questions, we used broad criteria to collect relevant items at the intersection of the three domains of focus: ‘AI’, ‘hiring’, and ‘inequality’. We operationalized this via the combination of the following keywords: (1) *AI and algorithms*; (2) *human resource management, hiring, recruitment, work, employment* and *organizational* processes; and (3) *(in)equality, fairness, bias, stereotypes, prejudice* and *discrimination.* To ensure that we captured relevant pieces, we searched multiple databases, starting with Google Scholar, then ProQuest, Sociological Abstracts, Social Sciences Citation Index, Sage Journals, and Social Science Research Network, until no new items emerged. Our initial search resulted in 389 publications.

In the second step, we screened publications, using the Rayyan platform to allow for a blind screening process by our four-author team. We coded publications as: (1) ‘highly relevant’, (2) ‘somewhat relevant’, and (3) ‘least relevant’ to our focal questions (see Supplemental Material C, page 4 for details). Following majority agreement (three or more team members) of 75%, we included 212 ‘highly relevant’ publications (i.e. central focus on and sustained analysis of the intersection of ‘AI’, ‘hiring’ and ‘inequality’).

Our third step involved a detailed mapping of these 212 publications, noting discipline, key questions, concepts, and insights (see Supplemental Material A for the coding system). Using the combined information on the journal, the authors’ background and disciplinary affiliation, along with the keywords and the abstract, we identified four broad disciplinary clusters connected to: (1) computing and data sciences (CS); (2) human resource, management, and organization studies (HRMOS); (3) social sciences (SS); and (4) legal scholarship (LS). Given the complexity and novelty of emerging knowledge, we do not view these groupings as rigid, though we observed distinct characteristics in each cluster. For instance, CS research on AI is vast; we thus limited our focus to publications with a clear focus on the social implications of AI. HRMOS scholarship is very diverse, with applied and conceptual writings. SS are the broadest, most heterogeneous group, encompassing sociology, psychology, philosophy, and socio-technical orientations. LS offers more focused writing on specific legal questions. In a small number of cases where an item could potentially belong to more than one cluster (i.e. socio-technical perspectives which draw expertise from the CS and SS), we assigned it to the cluster that was most substantively relevant (see Supplemental Materials C for further details).

### Phase 2: Problematizing review

While a scoping strategy is essential for identifying this emerging multidisciplinary body of work, the value of a problematizing approach is that it critically assesses how knowledge is being constructed, unpacking the underlying assumptions that shape knowledge creation, and offering new insights and questions as a result. It therefore differs from familiar review approaches (e.g. integrative, systematic) that seek to offer a ‘representative’ description of knowledge, aiming instead to ‘re-evaluate existing understandings of phenomena, with a particular view to challenging and reimagining our current ways of thinking about them’ (Alvesson and Sandberg, 2020: 1297). We followed four-key principles of the problematizing approach: (1) using researcher reflexivity as a resource; (2) recognizing that ‘less is more’ by focusing on the most substantively insightful contributions; (3) ‘reading broadly but also selectively’; and (4) working to problematize rather than accumulate knowledge (Alvesson and Sandberg, 2020: 1300) (see Supplemental Material C for further details).

Reflexivity is a key element that drives problematization. Reviewers are not perceived as ‘neutral’ but active actors whose ‘intellectual resources’ and ‘paradigms and fashions’ are essential in analytical processes (Alvesson and Sandberg, 2020: 1295, 1297). Given the team-based nature of our project, we refined the problematizing review principles to incorporate processes of co-reading and dialogue, moving from individual to more collective forms of knowledge creation (Mauthner and Doucet, 2008: 977). Specifically, we conducted numerous layered co-reading exercises, assigning a primary reader to each discipline to allow the accumulation of expertise in the specific domain, and secondary readers who rotated across disciplines, allowing for the generation of cross-disciplinary ‘bridges’. The depth and reflexivity that this type of co-reading enabled were essential to developing insights within and across disciplines concerning the key questions, concepts, and assumptions in the scholarship. These insights became a foundation for problematizing.

Our strategy of assigning a ‘primary reader’ to each disciplinary cluster to accumulate knowledge and a ‘secondary reader’ to rotate across disciplines fostered expertise in Phase 1 that allowed us to refine the initial corpus and target a more select subset of relevant publications in Phase 2. Following the principle of ‘less is more’, we created a ‘supercorpus’ – a selection of 97 readings, from the scoping review (57 items) and additional readings from reading ‘broadly but selectively’ – beyond the corpus (40 items). We identify items cited from this final corpus in our bibliography with an asterisk. A summary of the corpus, with cluster, key foci and key concepts appears in Supplemental Material B.

To identify scholarship for the ‘supercorpus’, we relied on each reviewer’s accumulated intradisciplinary knowledge, developed through immersive reading and engagement with their focal area. Our team’s collective intradisciplinary knowledge thus functioned as a form of quality assessment. Our selection criteria were designed to identify publications that: (1) offered the most substantive analysis, (2) sparked key debates, (3) introduced novel concepts, and/or (4) demonstrated high relevance to the intersection of AI, hiring, and inequality. In line with Alvesson and Sandberg (2020: 1297), we gave priority to conceptually generative insights. We also read beyond the initial corpus – a common practice in the problematizing method – by hand searching for key classic and contemporary texts to aid our analysis. While in Phase 1, our selection criteria were strongly tied to the intersection of three domains (i.e. AI, hiring, and inequality), in Phase 2, other items in neighbouring or overlapping domains (e.g. digitization) were considered to be relevant for a deeper understanding of current debates (further details appear in Supplemental Material C, page 7).

We then reviewed these 97 items using deep, reflexive, layered reading. This follows Alvesson and Sandberg’s (2020) view of problematizing as ‘an “opening up exercise” that enables researchers to imagine how to rethink existing literature in ways that generate new and “better” ways of thinking about specific phenomena’ (1290). We use Acker’s (2006) classic work on inequality regimes as a valuable framework for rethinking the potential of AI to contribute to organizational inequalities. We also incorporate contemporary scholarship on the broader implications of AI and digitization, including Elliott’s book (2019) on the growing use and cultural acceptance of AI, Zuboff’s work (2019) on surveillance capitalism, data, privacy and control, and Pasquale’s (2015) insights on the intentional complexity and opaque nature of AI, all of which set the stage for organizational use of AI.

In the following sections, we discuss questions and concerns examined across various disciplines (RQ1) and uncover underlying assumptions within different disciplines (RQ2). In the discussion section that follows, we problematize and explore novel ways of conceptualizing AI’s role in hiring, situating the analysis within Acker’s (2006) framework of inequality regimes. We conclude by considering the implications of our findings and offering an agenda for future research. Table 1 (below) summarizes our key findings.

**Table 1.** Summary of key findings and insights.

Discipline	Question 1: Key questions examined	Question 2: Underlying assumptions	Integration: Key insights & future research
CS	• Developing AI for efficiency and fairness in hiring. • Measuring and mitigating bias (e.g. demographic parity). • Debiasing datasets & algorithms. • Procedural justice in AI development and deployment.	• Bias originates from data and algorithms. • Technical solutions can address known biases but may fail to eliminate latent bias.	**Key insights:** • Emerging multidisciplinary discussion but with asymmetries in how key concerns are conceptualized. • Clear, heightened, potential for AI to conceal inequalities in hiring processes. • Contestation and lag over regulation and the ‘chain of responsibility’.
HRMOS	• Using AI to enhance efficiency, speed, and fairness in hiring. • AI’s role in automating vs. augmenting human decision-making. • Optimal collaboration between AI and humans in hiring processes.	• Organizational complexity and human bias necessitate AI. • AI-human collaboration is the future of hiring processes. • Technological change is a naturally occurring process.	
SS	• AI’s impact on social inequalities and discrimination. • Surveillance and data use in hiring • How AI is deployed in organizations • Regulatory frameworks for responsible AI use.	• Inequalities are systemic and deeply contextual. • Technical solutions alone cannot address structural inequalities.	
LS	• Legal frameworks for addressing AI bias and discrimination. • Adequacy of existing laws and need for proactive regulation. • Accountability and fairness in algorithmic decision-making.	• Existing legal frameworks may be inadequate to address algorithmic bias. • Proactive, multi-party approaches are required.	

## Results: The state of the multidisciplinary field

### RQ1. What key questions and concerns are being examined in distinct disciplines about the role of AI in organizational hiring and recruitment processes?

Our review begins by examining key questions posed across disciplinary clusters, recognizing the central role questions play in shaping knowledge production (Alvesson and Sandberg, 2000: 1294). While questions about bias, fairness, discrimination, transparency and accountability are shared, disciplines differ in how these issues are defined and the solutions they propose. CS emphasizes technical causes and fixes of ‘bias’; HRMOS focuses on trust and improving the hiring process; and SS and LS raise broader social, ethical, regulatory, and political questions. We begin with the CS literature, which drives the technological phenomenon we are studying and dominant ways of framing the problem.

***Computing and data sciences (CS)***: Broadly speaking, CS focuses on technical solutions (Lepri et al., 2018) and, in some cases, the social-technical interface (Amershi et al., 2014). The core interest is in developing AI to enhance organizational efficiency, accuracy, and authenticity in hiring, and measuring and mitigating bias (Glymour and Herington, 2019; Kuhlman et al., 2020). CS examines questions of inequality through the lens of ‘bias’, defined as systematic deviation between expected and actual values of the predicted outputs of an AI application, resulting in unequal (dis)advantage for individuals or groups (Kordzadeh and Ghasemaghaei, 2022). The concept of *demographic parity* (i.e. proportional representation of different groups) is commonly used as ‘ground truth’, with any deviation in AI algorithms or outcomes considered ‘bias’ (De Alford et al., 2020; Geyik et al., 2019).

Specific questions of interest in CS thus focus on technicalities regarding: (1) how to select and debias datasets used for training AI algorithms; (2) how to optimize AI algorithms to reduce bias in AI outputs (e.g. feature removal from models) (Geyik et al., 2019); (3) who should be involved in the debiasing process (e.g. AI developers, clients, end-users) (Amershi et al., 2014; Birhane and Cummins, 2019; Raghavan et al., 2020); (4) why bias mitigation techniques fail (Gonen and Goldberg, 2019); and (5) if it is not possible to eliminate bias, how AI developers can instead focus on ‘procedural justice’, ensuring that AI development and deployment processes are fair, transparent, and accountable (Lepri et al., 2018).

Organizations are a frequent focus of empirical investigations in CS for several reasons. First, they are sites of high-stakes AI deployment, where decisions can significantly impact individuals and risk breaching anti-discrimination laws (Baer, 2019). Second, organizational settings reveal visible forms of bias, such as disparities in workforce composition, wages, and job satisfaction (Lee et al., 2015). Finally, they offer accessible data for AI training and bias analysis. Yet, organizations are often treated in a generic manner, with limited attention to their specific dynamics, structures, or processes.

***Human resource, management, and organization studies (HRMOS)***: Much writing in this stream focuses on the practical application of AI tools in recruitment and selection, and the potential for more ‘efficient’ and ‘effective’ organizational processes as a result (Allal-Chérif et al., 2021; Upadhyay and Khandelwal, 2018). A first set of questions concern whether and how AI solutions, such as video interviews, chatbots, and automated screening tools, can improve hiring processes, making them faster, less costly, more predictable and less biased (Dattner et al., 2019; Newman et al., 2020). AI is often perceived as a solution to human bias, frequently conceptualized as ‘implicit’, suggesting the unintentional bias generated as a result of the complexity involved in human decision-making.

A second set of questions concern how AI can address uncertainty and complexity in organizational decision-making (Dwivedi et al., 2019). Debate here focuses on whether AI will replace (*automation*) or complement (*augmentation*) human decision-making (Dwivedi et al., 2019; Jarrahi, 2018; Raisch and Krakowski, 2021). There is a strong argument for AI-human collaboration, with AI handling routine tasks such as resume screening, while humans tackle higher-order, tacit knowledge tasks (Bankins et al., 2024; Dwivedi et al., 2019; Jarrahi, 2018; Shrestha et al., 2019). Huang and Rust (2018) suggest AI handles mechanical and analytical tasks, leaving intuition and empathy to humans. These discussions, however, rarely consider the ethical aspects of such collaboration (Bankins et al., 2024).

Trust is a third concern: that is, whether AI-based decisions are trustworthy and trusted within organizations, by applicants, and by the public, and what conditions can enhance perceptions of fairness. While AI promises to reduce human bias, applicants prefer and trust human ‘two-way’ interactions over machine-based ones (Acikgoz et al., 2020; Newman et al., 2020). HRMOS explores solutions to enhancing trust, emphasizing transparency, fairness, and algorithmic oversight (Langenkamp et al., 2020). A key concern is how algorithms trained on non-representative or skewed datasets create inaccuracies and bias (Tambe et al., 2019). Ethical and legal concerns are also raised, including privacy issues when using training data without consent (Dattner et al., 2019) and questions about which parties – developers or employers – bear responsibility for ethical outcomes (Martin, 2019).

***Social sciences (SS)***: Encompassing diverse fields, this scholarship provides a broader critical perspective on AI and hiring, incorporating varied theories and levels of analysis. One important area of interest is on *affordances* – that is, what AI enables in hiring. For instance, Ajunwa and Greene (2019) describe how automated hiring platforms facilitate the fungibility of workers within and between organizations, capturing and structuring data in ways that aid employer control and standardized management. Another issue concerns the bundling of diverse data (e.g. employer records, social media, personality assessments, behavioural traces) and heightened potential for novel forms of hiring discrimination (Cruz, 2024; Rosenblat et al., 2014; Zuboff, 2019). For instance, Cruz (2024) shows how AI and social media are used to collect and assess non-skill related information, such as political leanings or the willingness to relocate, highlighting concerns over privacy, bias, and discrimination.

If emerging technologies afford managers and employers enhanced power – through access to more information, sophisticated surveillance, and advanced analytics – what are the implications for workers and job seekers? Researchers are beginning to document how and whether discrimination occurs in algorithmic hiring and evaluation (Ajunwa, 2020b), and how individuals perceive and respond to these technologies (Gelles et al., 2018; Lee, 2018). Individuals may engage with, evaluate, or resist these systems. Studies raise concerns on how workers perceive and react emotionally to algorithmic hiring and management systems with attention to perception of fairness and trustworthiness (Gelles et al., 2018).

Given these concerns, there is also attention to what can or should be done to regulate new technologies or use them in ways that promote socially desirable outcomes. Some propose frameworks to mitigate bias through technical design improvements in AI systems (Lin et al., 2020). Others advocate for stakeholder engagement, such as the ‘Design for Values’ approach, which promotes designing socio-technical systems that support human rights (Aizenberg and Van Den Hoven, 2020). Unions and worker representatives are also recognized as key actors in protecting or securing rights, given their role within many workplaces (Todoli-Signes, 2019). Broader organizing efforts – beyond specific workplaces – are increasingly seen as crucial for shaping the development and use of AI technologies in ways that safeguard worker interests (Crawford et al., 2019). Legal frameworks are another focus in addressing the challenges posed by AI technologies (Rovatsos et al., 2019), particularly in relation to human rights, privacy, and data regulation. These concerns bridge SS and LS, particularly in employment, where non-discrimination is a core legal protection in many capitalist democracies.

***Legal scholarship (LS)***: Central areas of attention include bias, discrimination, transparency, and privacy, with particular attention to the potential harms faced by individuals navigating ‘opportunity markets’ (Ajunwa, 2020a; Kim, 2020). These concerns intersect with broad debates over AI global governance, such as the three competing models discussed by Bradford (2023) in *Digital Empires*: the US market-driven approach, which prioritizes corporate interests; the EU rights-driven model, emphasizing individual protections; and the Chinese state-driven model, focused on centralized control. Most studies in our review focus on US and EU contexts, reflecting the dominance of a Global North perspective and exclusion of the Global South. In the United States, legal attention typically centers on employment law, particularly Title VII (The Civil Rights Act of 1964), which prohibits discrimination on protected grounds, and the lack of proactive measures and limitations of complaint-based approaches. In contrast, writing on the EU emphasizes its preventative, rights-based approach. For example, the General Data Protection Regulations (GDPR) protects personal data, restricts solely automated decision-making, and mandates human oversight in high-stakes areas like hiring (Kaminski, 2018) – principles reinforced and extended in the EU’s 2024 AI Act, which we discuss later.

A recurring question in LS is whether existing frameworks are a match for rapidly evolving technologies that make it difficult or impossible to detect bias (Ajunwa, 2020a; Hacker, 2018; Kim, 2020). Bent (2020) questions whether laws ‘developed to govern humans’ can ‘translate readily to the government of machine-assisted decisions’ (805). Mann and Matzner (2019) warn that ‘with increased algorithmic complexity, biases will become more sophisticated and difficult to identify, control for, or contest’ (1). Ajunwa (2020a; 2020b) underlines how algorithms can radically amplify bias, extending the traditional scope of harm and affecting vast numbers of people.

Such concerns are accompanied by specific questions about how established law might address algorithmic bias and discrimination. In the United States, scholars debate whether legal concepts of ‘disparate treatment’ and ‘disparate impact’, and complaint-based versus proactive approaches, can inform evolving practice. If biases are unobservable and unknown, making complaint-based processes impossible, is proactive regulatory intervention at the design stage not essential (Kim, 2020)? Does failure to consider disparate impact constitute evidence of discriminatory intent and should proactive auditing approaches be mandatory (Ajunwa, 2020a)? Does achieving ‘fairness’ in employment decisions by accounting for protected characteristics constitute ‘algorithmic affirmative action’ and, if so, is this legal (Bent, 2020)? And since humans are involved in AI production and use, who is legally accountable for the inequalities that algorithms produce, given evolving relationships between employers, developers, vendors, and platforms (Ajunwa, 2020a; 2020b)?

Across jurisdictions, broader field-level questions emerge about stakeholder roles and governance, helping to connect key questions within this review. Do effective responses to algorithmic bias require ‘technical’ fixes (e.g. debiasing algorithms), legal reforms (e.g. improving existing anti-discrimination legislation), human oversight (e.g. third-party auditors), or a combination of socio-legal-technical solutions (Bornstein, 2018; Nachbar, 2021)? How should countries balance individual rights, business necessity, and systemic regulation (Kaminski, 2018)? And given the limitations of existing employment and labour law, what other areas of law – for instance, privacy law and consumer protection – might be mobilized into a multi-pronged legal approach (Hacker, 2018; Mann and Matzner, 2019)?

### RQ2. What are the underlying assumptions and approaches within different disciplines concerning algorithmic hiring and its relationship to organizational inequalities?

Building on our hybrid review approach, we problematize to think critically about the implicit / explicit assumptions and established conventions in each field. Overall, we find that disciplinary assumptions vary notably. CS assumes bias is a technical problem that can be isolated and solved; HRMOS assumes AI can ultimately help to improve human decision-making; SS assumes AI is entangled with broader systems of inequality; while much LS assumes AI can be governed through some type of legal regulations.

***Computing and data sciences (CS)***: A distinguishing feature of CS is its limited substantive engagement with organizational contexts and processes. Typically, organizations are viewed as data sources and ‘high-stakes’ domains that can elevate the importance of AI technical developments / applications. Despite this narrower lens, CS makes clear assumptions about how AI contributes to both the (re)production and mitigation of organizational inequalities. Specifically, it assumes the two key sources of bias are: (1) data and (2) algorithms (i.e. the methods used to process data).

For the former, issues such as inherent bias in the data used for algorithm training are core to the computing literature – specific aspects include data scarcity or missing data on underrepresented groups (e.g. racial minorities) and imbalanced / biased data representation of different social groups (e.g. the under-representation of racial minorities in training data) (Favaretto et al., 2019; Kuhlman et al., 2020). Such issues lead to further disadvantages for marginalized groups in AI applications such as CV screening and AI-powered job interviews and competency assessments (Geyik et al., 2019; Langenkamp et al., 2020).

Algorithms are often seen as the primary means for rebalancing and rectifying biases in training data. Common techniques include feature removal (e.g. excluding race as a predictor of performance in job applicant assessments), data reweighting (e.g. increasing the weighting of under-represented groups), and imposing external rules for calibrating algorithm outputs (e.g. the four-fifths rule whereby the proportion of candidates from a minority group should not be below 80% of the majority group) (Raghavan and Kim, 2023). However, some CS studies argue that algorithms can only address a limited number of known biases (i.e. characteristics such as gender and race explicitly chosen by users / designers) and cannot ‘remove’ biases embedded in complex latent patterns in training data that are not known or specified as a source of bias (Gonen and Goldberg, 2019). In short, algorithmic solutions cover up rather than remove biases that lead to AI-induced inequalities (Gonen and Goldberg, 2019). Recognizing that ‘seeing’ and ‘knowing’ biases are key to identifying and mitigating them, CS literature advocates for more diversity in the AI workforce (Kuhlman et al., 2020).

***Human resource, management, and organization studies (HRMOS)***: Though often focused on AI implementation in hiring processes, scholarship in this stream reflects several underlying assumptions. First, it is assumed that organizational processes in general, and hiring processes and decision-making in particular, involve vast complexity and uncertainty (Tambe et al., 2019). Second, human bias is assumed to be a product of this complexity, with human cognitive processes using biased shortcuts to handle complexity (Jarrahi, 2018). Third, technological change is assumed to be a naturally occurring process, impacting society and organizations (Black and Van Esch, 2020; Dwivedi et al., 2019).

Building on these assumptions, HRMOS writing often views AI through an optimist lens, offering a potential solution to organizational complexity, human bias, and changes in hiring due to increasing digitization and online job postings. Using the term ‘digital recruiting’, Black and Van Esch (2020) trace the shift from *analog hiring* (mostly manual) to *digital recruiting* (digitized and automated practices transformed and dominated by AI), portraying AI solutions as the ‘natural’ technological response to the massive outreach enabled by digitized hiring. Following this logic, AI is seen as the inevitable robust solution to the proliferation of job applications. Thus, we see a large consensus in HRMOS on the future being identified by ‘human-AI’ collaboration (Einola and Khoreva, 2023; Jarrahi, 2018; Kelan, 2023; Shrestha et al., 2019). Reflecting these assumptions about the future, a special focus within HRMOS literature is on conceptualizing how this collaboration will unfold, and how specifically it will help to deal with uncertainty, complexity, and human bias.

For example, Jarrahi (2018: 7) develops a model of ‘human-AI’ symbiosis that can aid the ‘uncertainty’, ‘complexity’ and ‘equivocality’ of the decision-making process. Humans are seen as better equipped to make choices and decide ‘where to seek data’, while AI can ‘collect, curate, and analyse’ the chosen data. Shrestha et al. (2019) highlight the need to consider decision-making conditions, such as the ‘specificity of the decision search space’, ‘interpretability of decision-making process and outcome’, ‘decision-making speed’, and ‘replicability of outcomes’ (67–68). This approach underscores the strengths of AI, such as the speed and capacity to analyze large data, while human decision-making offers better interpretability and a more loosely defined decision space.

A strong underlying assumption in the literature is that tasks associated with recruitment and selection are particularly complex and, therefore, particularly challenging to automate (Tambe et al., 2019). Furthermore, various tasks involved in these processes are assumed to have different levels of complexity and, therefore, require different types of expertise and knowledge. While some tasks in hiring are routine and can be easily automated (Upadhyay and Khandelwal, 2018), others require intuition and tacit knowledge of humans (Jarrahi, 2018). Bankins et al. (2024) use the terms ‘data and AI sensitivities’ and ‘task sensitivities’ to describe the compatibility between different task characteristics with humans or AI in the particular organizational context (843).

Critical voices are also evident in the HRMOS scholarship, questioning some of these optimistic assumptions. Though more of a minority view, these perspectives rest on different assumptions: that AI raises moral and ethical considerations (Budhwar et al., 2022: 1084) and must be used as a socially responsible tool (Chang and Ke, 2024); and that AI adoption may be more complex than anticipated, given that job seekers prefer human interaction (Acikgoz et al., 2020; Newman et al., 2020) and that algorithmic decisions are perceived as fairer if humans have the final say (Newman et al., 2020; Tambe et al., 2019).

***Social sciences (SS)***: There are many strands of research, approaches, and assumptions in this stream – rooted in sociology, psychology, and science and technology studies, among others. Studies also address varied levels of analysis (e.g. micro, meso, macro). That said, one core, seemingly shared, assumption is that algorithms pose concerns for existing social inequalities, with the potential for mimicking, amplifying, or contributing to their new forms (Chen, 2023b; Lin et al., 2020).

These assumptions are linked to the social implications of technological change. While some research suggests that technological change unfolds in a way that is somewhat predictable (i.e. we assume that computers continue to operate in a certain way), others suggest a fundamental break in the way technology is developing (Crawford et al., 2019; Elliott, 2019). Some scholars view technology as a deeply embedded socio-technical system, in which humans and technologies shape one another (Wajcman, 2017), whereas others view technology as an external force imposed on workers (Ajunwa, 2020b; Zuboff, 2019).

Building from this, a significant part of the SS scholarship problematizes the technosolution framework stemming from the computational perspective to algorithmic inequality (Drage and Mackereth, 2022; Osoba et al., 2019; Rovatsos et al., 2019). Social perspectives underscore that the complexity of inequality cannot be reduced to data and its calibration, such as challenging the removal of demographic attributes from the algorithms’ training as a key method of ‘debiasing’ (Drage and Mackereth, 2022). Removing protected characteristics from AI training and design is seen to misfocus on the category itself rather than the systems of power that are responsible for the differential treatment of these groups.

Furthermore, understandings of equality, equity, and fairness are deeply contextual and may vary across different settings (Osoba et al., 2019; Rovatsos et al., 2019), posing a challenge for the technical and computational universalist discussion of ‘debiasing’. For instance, Osoba et al. (2019) point out that the definitions of *procedural equity* (related to the fairness of the decision-making process) and *outcome equity* (related to the fairness of the outcome) mean different things in different contexts. Overall, the SS perspectives point to the systemic nature of inequalities, including algorithmic, that require structural, rather than individual-level and/or technical perspectives (Joyce et al., 2021).

***Legal scholarship (LS)***: Legal scholars hold diverse views on the challenges of algorithmic hiring and decision-making. While some scholars view the foundational issues as legal (Ajunwa, 2020b; Bornstein, 2018), others emphasize technical dimensions (Blass, 2019). Many scholars adopt either implicitly or explicitly some form of socio-technical-legal approach, recognizing the complexities and need for multifaceted solutions that transcend the boundaries of law. Overall, three sets of assumptions, or debates over these issues, stand out.

First, we see varied assumptions around the efficacy of existing legal frameworks to address potential problems of algorithmic bias and discrimination. Some argue they are up to the task (Nachbar, 2021), while others advocate for change (Ajunwa, 2020a). Secondly, while legal scholars are doubtful of the argument that algorithms minimize bias (Bent, 2020), many advocate for ‘technical fixes’ in data and design as the best approach for mitigating bias. Finally, some scholars assume that the nature of algorithmic bias requires more proactive, multi-party approaches (e.g. third-party auditing), noting that complaint-based employment laws are insufficient (Ajunwa, 2020a; Hacker, 2018; Kim, 2020).

Building on these assumptions, legal scholars propose a variety of approaches for promoting fairness and mitigating discrimination. In the United States, Ajunwa (2021a) advocates for a proactive approach that prevents bias at its source by mandating alterations to data inputs and implementing third-party certification processes involving legal, software engineering, and data science expertise. Bent (2020) explores the legalities of incorporating protected traits into algorithm design, advocating for ‘algorithmic affirmative action’ (809). Blass (2019) focuses on data protection, proposing a third-party recordkeeper system to safeguard representative data. Bornstein (2018) advocates for an approach with proactive and reactive dimensions that involves regulating and documenting algorithmic choices before use, but also strengthening existing law to address algorithmic disparate impacts post-occurrence. This dual approach aims to improve algorithms and legal frameworks simultaneously.

More integrated legal frameworks, particularly within the EU, offer a distinct avenue for combating algorithmic bias (Parviainen 2022). Hensler (2019) highlights the merits of the GDPR, while advocating for proactive regulation to audit algorithms for discriminatory effects. Kaminski (2018) suggests a binary approach, combining individual due process rights with collaborative governance for systemic regulation. Mann and Matzner (2019) emphasize the importance of EU regulations in protecting individual rights, especially data privacy and the right not to be subject to automated decisions. They also offer unique insights into less discussed issues of intersectional discrimination (e.g. gender × race), emergent discrimination (e.g. browsing histories), and the need for decolonial perspectives to understand how algorithmic profiling may perpetuate colonial legacies. These latter points echo Acker’s (2006) emphasis on the need to centre globalization and inequalities.

## Discussion and critical reflections

### Problematizing and identifying new questions and concerns

Building on these findings, and deepening our problematizing approach, this section brings together multidisciplinary scholarship to identify novel questions and concerns about the challenges posed by AI-mediated hiring. First, in tracing multidisciplinary conversations about AI, hiring, and organizational inequalities, we note emerging asymmetries in how knowledge is being constructed and the dominance of concepts and approaches, especially from CS. Second, in examining hiring processes as foundational gatekeeping mechanisms that structure access to jobs, we note how AI is becoming intertwined with the ‘inequality regimes’ in organizations, concealing inequality further within AI’s opaque apparatus. Finally, we note a paradox between stated concerns over AI, regulatory lags, and contestation. Analytically, we situate these trends within critical perspectives on power asymmetries and the role of AI in both exacerbating and concealing them further. We conclude by discussing an agenda for future research and each stakeholder’s imagined role in the chain of institutional and collective responsibility (Miller, 2017: 344).

**Figure 2.**
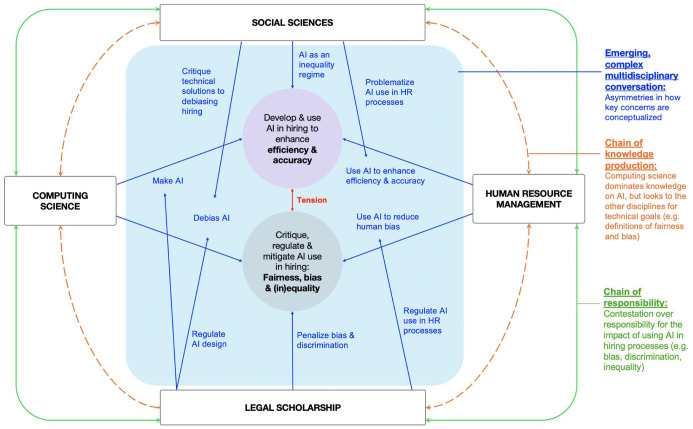
Review synthesis – Interconnected dynamics across disciplinary domains.

Figure 2 provides a synthesis of our review and visualizes the complex interplay across disciplinary domains. In the centre, the blue lines indicate interdisciplinary conversations and the interrelations of key questions and concerns across the disciplines. The dotted orange lines illustrate the chain of knowledge production across disciplines, such as asymmetries in emerging knowledge. The green lines indicate emerging strains within the chain of responsibility across the domains, such as contestation over the responsibility for the consequences of AI usage. We discuss each of those in turn in the subsections below.

***Tracing asymmetries in emerging multidisciplinary knowledge***: While all disciplinary perspectives are concerned with the potential of AI to create / reproduce inequalities in hiring processes, each brings unique assumptions and questions. CS focuses on technical solutions and adjusting data and algorithms to improve performance. HRMOS advocates for implementing AI, and advancing human-AI collaboration, to gain efficiencies and overcome problems of human bias. SS scholarship brings diverse and often critical perspectives to bear, discussing how new technologies are developing within the broader social context and their potential risks for (re)producing inequalities. LS focuses on the efficacy of varied legal and regulatory solutions.

Bringing different disciplinary perspectives together allows us to identify shared concerns and, more importantly, clear asymmetries in emerging knowledge, with intellectual spillover in concepts, questions, and concerns. CS is clearly dominating the discussion through expert knowledge of the subject matter: the algorithm. This is evident in the escalating presence of CS publications and perspectives within the multidisciplinary scholarship we traced, but equally important in other disciplines through the adoption of computing terminology and mathematical definitions, such as ‘algorithmic bias’, ‘algorithmic fairness’, and ‘debiasing’. A problematizing approach illuminates how knowledge is embodied in language and how terminology is shaping how ‘algorithmic inequality’ is conceptualized. This tendency has several implications for knowledge generation.

First, the widespread adoption of the terms ‘bias’ and ‘debiasing’ across disciplines contributes to discussing the issue from the technosolution perspective that assumes that complex inequality can be resolved technically (with proper data, design and interpretation of the outcome) to achieve *algorithmic fairness*. Yet, designing a ‘fair’ algorithm is difficult, if not impossible, given the contradictory nature of fairness criteria (Madaio et al., 2022; Osoba et al., 2019; Rovatsos et al., 2019). Scholars note tensions between *anti-classification* (hiding the protected characteristics), *classification error parity* (ensuring that chances of positive outcomes and errors are the same), and *calibration* (ensuring that risk scores are resulting in the same percentage outcome) (Rovatsos et al, 2019). Moreover, the meaning of fairness is deeply contextual and should be defined / operationalized in a domain-specific manner (Osoba et al., 2019). Yet, in most discussions, the CS perspective goes unchallenged.

Second, we identify a recurring tendency in CS to focus on ‘debiasing’ while looking to other disciplines for guidance on issues of justice, fairness, and equality in hiring (Chen, 2023a; De Cremer and De Schutter, 2021), or alternatively to delegate such issues away (Belenguer, 2022). Although some computational studies in our review do engage with other disciplines, it is more often a utilitarian than conceptual exchange. Many studies seek practical suggestions on how to improve the data, the model, and / or the outcome to achieve demographic parity between different groups. Thus, we see the adoption of the four-fifths rule – a core principle in US employment law – as a widely accepted ground truth in algorithm development and testing (Raghavan and Kim, 2023). Such ‘outsourcing’ reaffirms the centrality of a ‘technosolution’ paradigm, which in turn results in a decontextualized approach to AI-induced inequality – another observation of our review.

Decontextualization occurs in several ways. First, the populations from which AI training data are drawn are rarely discussed, including whether these populations can be representative of other populations for which the algorithms are to be deployed. Thus, the *transferability* of data across contexts, groups, and populations – a critical issue in a globalized economy – is unclear (Yu, 2020). Second, the organizational settings for which algorithms are developed are rarely discussed. Yet, these settings vary widely, depending on industry, labour force composition, company size, location, and relevant local, national, and global laws. Such contextual knowledge is actually critical for reconciling contradictory rules to achieve ‘algorithmic fairness’ (Osoba et al., 2019) and determining whether algorithmic inequalities will be minor or significant (Rovatsos et al., 2019).

***Tracing how algorithms are intertwined with ‘inequality regimes’***: Expectations of technical solutions to resolve issues that are broad, pervasive, and concealed, illustrate the current influence of a technosolution paradigm. Yet, within our multidisciplinary review, other perspectives critique the expectation that algorithms can resolve issues that (re)produce inequalities via proper data and calibration as being overly simplistic and optimistic (Drage and Mackereth, 2022; Kelan, 2023; Köchling and Wehner, 2020). The framing of algorithmic tools as solutions to human bias (Lin et al., 2020; Upadhyay and Khandelwal, 2018) implies that inequalities are a problem occurring at the *individual level* (e.g. human / implicit bias), overlooking their *structural* pervasiveness and embedding in organizational contexts.

Taking a problematizing approach to these issues, we argue that both human and algorithmic decision-making processes are situated within structural *inequality regimes*, reproducing relations of power in ways that are visible and invisible (Acker, 2006). Within organizations, status group inequalities are created and/or reinforced, through segregation or integration, or the granting of (un)equal access to resources or other forms of power (Ozturk and Berber, 2022; Ray, 2019; Tomaskovic-Devey et al., 2009). These inter-group dynamics reflect power relations rooted in specific economic, social, and legal contexts, at local, regional, national, and global levels. Furthermore, organizational processes are not isolated from broader power dynamics. As Pasquale (2015) argues, the proliferation of opaque, algorithmic systems reinforces these dynamics by concealing decision-making processes further, thus limiting visibility and accountability.

Specifically, the complex and opaque nature of algorithmic solutions (Elliott, 2019; Kellogg et al., 2020; Pasquale, 2015), lack of transparency (Langenkamp et al., 2020), and ability to easily incorporate explicit and implicit information about job applicants (Cruz, 2024) contribute to further invisibility of differential treatment (Acker, 2006), hidden within the apparatus of automated or augmented decision-making (Raisch and Krakowski, 2021). Building on Pasquale (2015), Ajunwa (2020a: 1724) warns that automated hiring systems ‘may become the worst type of broker, a “*tertius bifrons*”’ between the employer and employee, cementing algorithmic authority over hiring processes and sustaining a new, algorithmic, ‘black box’. This renders decision-making more opaque, inequalities more concealed, and job seekers more powerless – signalling the emergence of an *algorithmically-mediated* inequality regime. This regime is characterized by extreme levels of *algorithmic invisibility*, as well as pervasive and unchallenged *algorithmic legitimacy*.

Data play a key role within algorithmically-mediated inequality regimes. Data are used to feed and train algorithms, which in turn, collect and analyze data, often without proper consent (Dattner et al., 2019). Operating as a part of an algorithmic system, data extend beyond the required application materials to include information collected indirectly (e.g., datafied social media profiles) that may signal protected characteristics and behavioural traces. Both direct and indirect data can serve as filters for inclusion or exclusion (Cruz, 2024; Todoli-Signes, 2019). Zuboff (2019) refers to behavioural data as the new ‘gold dust’ of surveillance capitalism, offering huge predictive and economic value (Todoli-Signes, 2019). The use of online data is an increasingly contested area, where practices are difficult to regulate, not entirely illegal but potentially unethical, creating an invisible space for inequalities to occur and reproduce (Rosenblat et al., 2014; Zuboff, 2019).

The expanded use of datafied labour profiles (e.g., resume databases, LinkedIn pages, algorithmic matching scores) illustrates this concern. In traditional hiring, applicants choose how to present and narrate themselves. In algorithmic hiring, the unrestricted access to digital data shifts control away from applicants, toward opaque algorithmic systems that collect and use digital traces in ways that are increasingly (*algorithmically*) invisible (Ajunwa, 2020a; Pasquale, 2015). As employers derive power from access to more data, candidates have less power and less agency. Yet, attempts to legitimate current practices are made through claims of the unprecedented volume of digital job applications, illustrating the emergence of (*algorithmic*) legitimacy that references the necessity of algorithmic solutions despite contestation and concerns. These claims of ‘algorithmic necessity’ shift the blame onto job seekers as the ones who generate massive flows of applications while, in fact, the gatekeeping power is concentrated with the employers, who can potentially benefit via an enlarged pool of applicants and data. Candidates respond to digital hiring using the platforms, but for them this means increased competition, minimized chances of being hired, and less control over the process.

Another illustration of *algorithmically-mediated inequality regimes* can be seen in the use of facial recognition to evaluate candidates. Framed as tools of efficiency, facial recognition systems measure, code, and interpret candidates’ facial expressions through opaque algorithms that are nearly impossible to question, understand, or appeal (Ajunwa 2020a; Pasquale 2015). This makes the process of evaluation *algorithmically invisible*, and generates skewed, one-sided visibility, while employers see everything and candidates see little. Codified facial expressions are what Zuboff (2019) calls ‘behavioural surplus’, predictive data for employers to define which facial cues signify the ‘ideal applicant’. This strips candidates of agency over how to present themselves in the hiring process.

Given how pervasive AI in hiring processes is becoming, it can and will intertwine with many other decision-making processes, embedding itself throughout organizational encounters and interactions. This has several implications. First, highly specialized expert knowledge is required to understand algorithmic tools, their design, and calibration (Pasquale, 2015). This knowledge is not widely shared by all stakeholders involved in the hiring process, confirming the emergence of a new technical ‘coding elite’ – stakeholders possessing exclusive knowledge on the design of algorithmic solutions (Burrell and Fourcade, 2021: 215). Second, and related to our first point, the lack of transparency and the need for elite knowledge pose challenges for regulation, accountability and responsibility (Martin, 2019; Miller, 2017), a point we return to shortly.

***Tracing questions of regulation, legitimacy, and accountability***: A final insight from our review highlights questions about the regulation of algorithmic hiring as well as contestation over how problems of unfair treatment and accountability will be addressed. Here, the LS and SS scholarship offers more critical perspectives, underscoring the need to accelerate regulation, and questioning (with some exceptions) the faith placed in purely technosolutions. That said, some legal scholars do argue that technical fixes, voluntary audit approaches, or a combination, are sufficient, with problems handled via existing legal frameworks. For instance, in the United States, Sonderling et al. (2022) argue against stringent regulations, proposing that federal agencies such as the Equal Employment Opportunity Commission (EEOC) provide expert opinion to clarify the law and engage companies in voluntary compliance.

While technological advancements continue to outpace regulation, frameworks are emerging under which the multiple stakeholders involved in algorithmic hiring – employers, vendors, contractors, computer scientists, workers, unions, advocacy and civil society groups – will operate. LS offers insights on the emerging regulatory landscapes as discussed by Bradford (2023) and others (see Table 2 below). Much attention focuses on the US model, which emphasizes efficiency and minimal government intervention. A smaller subset of EU-focused studies considers more ‘muscular’ models (Kim and Bodie, 2021), specifically the EU’s GDPR and the 2024 EU AI Act, which place strong restrictions on high-stakes decision-making like hiring, requiring risk impacts, mandatory pre-deployment, transparency measures, and human oversight (Kaminski, 2018; Kaminski and Malgieri, 2025). Yet, there are many questions about the impact of regulation and the potential for workarounds, such as human-led rubber stamping of algorithmic decisions to avoid illegality (Parviainen, 2022). Moreover, at the time of writing, lawmakers in the United States are considering bans on AI regulation at the state level (Lima-Strong, 2025). It is also notable that other regulatory models, such as the state-centred Chinese model that Bradford (2023) outlines, and others, are not discussed, confirming the dominance of Global North perspectives. Future research and comparative analyses across systems will be important for shedding light on how distinct modes of AI may contest one another in a global AI race. Indeed, this contestation may be crucial to understanding the role of AI in reproducing inequalities on a global scale.

**Table 2.** Summary of current regulatory approaches.

Dimension	United States	Europe (EU/UK)
Regulatory approach	Market-driven, complaint-based. Discrimination is addressed through litigation. Burden is placed on individuals to prove and contest discrimination.	Rights-based, preventative. Comprehensive regulation offers preventative safeguards. Responsibility for fair treatment lies with employers and regulators.
Key legislation and definitions	Anti-discrimination statutes (e.g. Title VII, EEOC) with some state / local AI-specific measures (e.g. NYC bias audits). Bias and un/fairness are established through measurable disparate treatment and impact (e.g. four-fifths rule).	The 2024 EU AI Act regulates ‘high-risk’ decision-making (e.g. hiring). It establishes broad definitions of fairness, and mandates risk impacts, data protection, mandatory pre-deployment, transparency measures, and human oversight of decisions.
Enforcement mechanisms	Reactive, with a few exceptions (e.g. NYC audits). Investigations and remedies depend on specific complaints and vary across jurisdictions. Without legal challenges, discrimination is left unaddressed.	Proactive. Regulators can demand evidence of compliance, impose conformity assessments, and levy significant fines. Current regulations seek to prevent harm, but enforcement may vary across member states.
Consequences for applicants	Limited rights: Applicants are typically unaware when algorithmic tools are used. The onus is on applicants and advocacy groups for litigation and complaints.	Stronger procedural rights: notification of AI use, access to human review, avenues for appeal. Applicants can contest unfair practices directly.
Organizational interventions	Voluntary in most jurisdictions. Proactive organizations can embrace privacy and data protection protocols, internal audits and/or third-party auditing, model documentation, and cross-functional fairness committees to identify and mitigate risk and ensure fair treatment.	Mandatory. Regulations require organizations to carry out risk assessments, data governance, and bias testing for high-risk AI. Organizations can also institutionalize ethics by design, participatory design approaches, and continuous post-deployment monitoring. These measures translate rights to fairness, transparency, and accountability into everyday HR practices.

Bringing the disciplines together helps pinpoint key challenges in regulating algorithmic hiring, given the myriad stages where discrimination can occur, the ways in which unfair treatment is concealed in ever more complex algorithmic ecosystems, and the limits of existing legal approaches (Osoba et al., 2019). Our review highlights diverse viewpoints, however, showing that the path ahead is not predetermined. This is illustrated in Table 2 below. In the United States, Title VII of the Civil Rights Act has been a key tool for combatting employment discrimination, prohibiting *disparate treatment* and *disparate impact*. But a serious problem with the US approach is the burden of proof placed on individual complainants, prompting calls for alternatives such as proactive audits, third-party certifications, and hybrid legal approaches that blend employment and consumer protection / privacy law. Developments such as New York City’s 2023 law require proactive audits to ensure fairness; employers must notify applicants when using these tools, provide other options, conduct annual audits, and publicly disclose results. Yet, loopholes – such as the inadequate independence for auditors – limit its effectiveness (Fuchs, 2023). US law also does little to aid transparency and accountability, given trade secret protections (Kim and Bodie, 2021).

Acker’s (2006) ideas are useful for thinking through the current challenges of regulation and algorithmic hiring. Historically, she observes, inequalities have been successfully challenged when their visibility is high and their legitimacy is low. Today, AI threatens to further obscure this visibility, making it harder, or impossible, to identify what is (re)producing inequalities. This *algorithmic* invisibility challenges existing regulatory frameworks that rely on the paradigm of redressing the differential treatment and outcomes caused and mediated by humans. Equally important, current regulatory lag and contestation, certainly in the market-based U.S. system, may be allowing legitimacy to grow around algorithmic hiring, positioning it as a ‘business necessity’, despite growing concerns.

A critical question, then, is how these *algorithmically-mediated* inequality regimes can be tempered and restrained, and what new legal paradigms are suited for this new organizational reality. Acker (2006) argues that successfully challenging inequalities in the past has required the combined force of the law, civil advocacy, and social movements outside of organizations, along with change agents inside organizations. This aligns with Zuboff’s (2019) assertion that democratic oversight over surveillance capitalism necessitates mobilized collective resistance where social movements can be seen as critical agents in challenging the asymmetries embedded in and reproduced by algorithmic systems. Our review underscores the urgent need for deeper, multidisciplinary engagement by scholars in these areas, alongside allied efforts by diverse stakeholders – for example, workers, unions, advocacy groups, and regulators – to ensure fair and accountable AI systems and their deployment.

### Contributions, limitations, and future research directions

Our review makes contributions to the emerging understanding of AI, hiring processes, and organizational inequality, with implications for: (1) theoretical understandings; (2) knowledge production; and (3) practical applications. Below we outline these contributions and propose future research directions, emphasizing the need for advancing theoretical understanding of AI as a part of inequality regimes, fostering interdisciplinary collaboration, and bridging gaps between academia, policy, and practice.

It is important to note that our review is limited to English-language publications, reflecting a Global North bias and under-representing perspectives from the Global South. Future research must prioritize comparative work across diverse jurisdictions and global perspectives to fully understand the complexities of algorithmic hiring. Moreover, while our hybrid methodology offers strengths in mapping and analyzing emerging multidisciplinary scholarship, publication practices and quality vary across fields (e.g. CS often publishes in peer-reviewed conference proceedings rather than indexed journals). Future studies may therefore wish to employ systematic review methodologies that include explicit quality measures. Finally, while our review focuses on hiring processes as a foundational gatekeeping mechanism, other HR decision-making processes altered by AI (e.g. promotions, compensation) merit attention in future research (Manroop et al., 2024).

***Theoretical understanding:*** By applying and extending Acker’s framework, we re-conceptualize AI-transformed organizations as sites of *algorithmically-mediated* inequality regimes. These regimes are reinforced through the growing legitimacy and pervasiveness of AI-driven solutions, and their capacity to conceal inequality further within the algorithmic mechanisms that require expert knowledge to be fully understood. Framing AI as a part of ‘inequality regimes’ enables a deeper understanding of how AI both participates in and transforms the structure of inequality within organizations.

Future research should continue to theorize AI’s role in perpetuating inequalities, advancing knowledge of AI-mediated mechanisms identified by the growing legitimacy and invisibility of high-stakes decision-making processes. Critical perspectives from varied disciplines (e.g. sociology, political economy, law, etc.) can offer valuable insights into whether AI emerges as a distinct structural mechanism that reproduces / conceals inequality, or interacts with and embeds in existing mechanisms. A particular direction for theorizing should consider the multilayered relations of power beyond the organizations (Acker, 2006), such as political, economic and international structures. Building on and extending the work of Zuboff (2019) and Pasquale (2015) could help to trace the ways in which a new economic order is spilling over and defining how AI is operating with high-stakes social institutions and its regulation. Specifically, it will be important to continue conceptualizing these tendencies within organizations – particularly in AI-mediated hiring – by examining whether and how AI systems are gaining legitimacy, shifting from optional tools to imposed necessities.

Studies grounded in qualitative traditions (e.g. ethnography, interviews, historical and comparative designs) will be especially helpful for exploring the discourses, practices and attitudes around AI, and understanding how AI may reproduce inequalities, experiences of algorithmic inequality, and efforts to mitigate it. Specifically, ethnographic studies can trace practices related to AI through different ‘standpoints’ (Smith, 2005), such as the practices of creating AI, focusing on the computing industry; the practices of using AI, focusing on domains of application (e.g. workplaces, healthcare, education, etc.); and the practices of regulating AI, focusing on legal and regulatory developments. Studying these topics can elucidate political, economic and other forms of power asymmetries, thus contributing further to conceptualizing the place and role of AI in shaping inequalities.

***Knowledge production:*** Our review highlights the key role of multidisciplinarity in generating and advancing knowledge about AI applications for social inequality. Here, we can extend the concept of ‘chain of responsibility’ (Miller, 2017) that frames a collective responsibility of different stakeholders in AI applications to knowledge production, highlighting that a ‘responsible’ understanding of AI requires *a chain of knowledge and expertise*. While various disciplines share a common concern for addressing social inequality, they work with distinct questions, concerns, and assumptions, as our review has shown. These disciplinary approaches typically confine analyses to domain-specific expertise and objectives, overlooking insights from other fields. For example, CS possesses the expertise on the subject matter, the algorithm, but has limited knowledge on the domains of its application in hiring, including the organizational contexts and constraints, relevant policies, and regulations. SS, HRMOS and LS each have expert knowledge of their domains, but typically lack expert knowledge to evaluate algorithmic solutions employed in their domains. These disruptions to the chain of knowledge obscure an understanding of how algorithmic solutions operate in and interact with high-stakes processes and decisions such as hiring. To address these gaps, there is a need for more meaningful engagement between disciplinary areas with implications both for *how* to collaborate and *what* to collaborate on.

In terms of *how* to collaborate, fostering more meaningful engagement between disciplinary areas is essential for achieving a true post-disciplinary approach to AI, one that is human-grounded, socially embedded, and that challenges the technosolution paradigm circulating around AI use and mitigation of its impact. Currently, CS dominates much of the current discourse. However, insights from SS, LS, and HRMOS studies are critical to understanding the organizational, institutional and structural forces in place. Collaborative efforts across these fields can lead to a more comprehensive understanding of what ethical and responsible AI can look like, fostering solutions that incorporate both technical and contextual, domain-specific, dimensions. Specific mechanisms that foster interdisciplinary research, such as joint conferences, funding initiatives, research networks or special issue calls, can facilitate collaboration across disciplines and sectors, allowing vital exchange between academics, algorithm designers, policy creators and legal experts.

In terms of *what* to collaborate on, our review highlights many pressing issues. First, the foundational concepts of algorithmic (in)equality, fairness, ethics, and transparency require rethinking, as current definitions often fail to address entrenched systemic inequalities and contextual characteristics. Second, a multi-level approach – spanning micro, meso, and macro – is crucial for developing frameworks that prioritize structural approaches to inequality in the organizational application of AI, with more awareness of the specific level at which research is conducted. Third, research on AI and hiring demands stronger contextualization to account for differences across industries, company size, and workforce compositions. For example, research could focus on implications of AI solutions for historic, complex, and intersectional inequalities. Fourth, governance of algorithmic hiring systems requires multidisciplinary research to ensure equity, feasibility, and accountability. Initiatives such as the EU AI Act exemplify the multidisciplinary and cross-sectoral approach to developing AI governance frameworks (Rotenberg and Kyriakides, 2025). We suggest a stronger scholarly focus on tracing how the *chain of knowledge* operates within such initiatives, examining how ideas, assumptions, and approaches cooperate.

***Practical application:*** Building on insights into the *chain of knowledge*, another priority is to operationalize *the chain of responsibility* at a practical level. As our review shows, algorithmic hiring systems can obscure structural inequalities while deflecting accountability across multiple stakeholders. Because challenges cannot be solved by a single stakeholder, we advocate for a collaborative agenda involving employers, organizations, algorithm designers, unions, regulators and policy makers, with each assuming and maintaining their unique responsibility in the chain. In terms of concrete steps, employers can play a critical role through participatory design practices and voluntary audits to ensure AI systems are transparent and fair. They can also build capacity and awareness on responsible AI use by fostering partnerships between HR professionals, algorithm designers, and regulators, to evaluate real-world AI applications, identify barriers to fairness, and embed these insights into their own hiring practices. Likewise, legal practitioners and scholars can work with policy makers to craft regulations that respond to the evolving challenges that AI presents. Interdisciplinary resources can bridge gaps between legal, policy, and organizational domains. Collaborative efforts must be tailored to specific contexts (e.g. industry, country), recognizing that equity and fairness are deeply contextual. One example is The *AI Policy Sourcebook* (Rotenberg and Kyriakides, 2025), a comprehensive handbook of AI policy frameworks from around the world, that provides a valuable practical tool for coordinated action.

It is important to recognize that power amongst diverse stakeholders is uneven and not all actors will choose, or be able, to act. Some employers may adopt responsible AI practices, others may not; regulators may intervene, or remain inactive. Civil society groups (such as Algorithmic Justice League, AI Now Institute, Upturn) will be critical in ensuring responsible AI practices, by engaging in public advocacy, submitting regulatory briefs, and educating job seekers about AI in hiring, while holding employers and developers accountable. As Zuboff (2019) and Pasquale (2015), and earlier Acker (2006), argue, their work is vital for making structures of inequality visible and creating pressure for change.

## Conclusions

Prompted by the growing use of AI in hiring, and concerns over organizational inequalities, this review combines scoping and problematizing approaches to assess emerging knowledge. We examine four broad disciplines – computing science; human resource, management, and organization studies; the social sciences; and law – that are largely siloed, tracing their key concerns and assumptions. Our review shows that (1) computing science’s dominance is driving attention to technosolutions, with the language of ‘bias’ spilling into other fields; (2) framing AI as a solution to human bias diverts attention from structural inequalities that are evolving into the *algorithmically-mediated* inequality regimes; and (3) regulatory responses are lagging, uneven, and contested, with solutions shaped by Global North perspectives. Our future research agenda emphasizes the need for deeper theoretical insights; an interdisciplinary *chain of knowledge* creation; and operationalizing the *chain of responsibility* for practical solutions. Building on Acker (2006), we stress that inequalities are most effectively addressed when visible and illegitimate – the opposite of what we currently see in the growing use of AI in organizational hiring.

## Supplemental Material

sj-docx-2-hum-10.1177_00187267251403902 – Supplemental material for Problematizing the role of artificial intelligence in hiring and organizational inequalities: A multidisciplinary review Supplemental material, sj-docx-2-hum-10.1177_00187267251403902 for Problematizing the role of artificial intelligence in hiring and organizational inequalities: A multidisciplinary review by Karen D Hughes, Alla Konnikov, Nicole Denier and Yang Hu in Human Relations

sj-docx-3-hum-10.1177_00187267251403902 – Supplemental material for Problematizing the role of artificial intelligence in hiring and organizational inequalities: A multidisciplinary review Supplemental material, sj-docx-3-hum-10.1177_00187267251403902 for Problematizing the role of artificial intelligence in hiring and organizational inequalities: A multidisciplinary review by Karen D Hughes, Alla Konnikov, Nicole Denier and Yang Hu in Human Relations

sj-pdf-1-hum-10.1177_00187267251403902 – Supplemental material for Problematizing the role of artificial intelligence in hiring and organizational inequalities: A multidisciplinary review Supplemental material, sj-pdf-1-hum-10.1177_00187267251403902 for Problematizing the role of artificial intelligence in hiring and organizational inequalities: A multidisciplinary review by Karen D Hughes, Alla Konnikov, Nicole Denier and Yang Hu in Human Relations
