# Supplementary material for: Problematizing the role of artificial intelligence in hiring and organizational inequalities: A multidisciplinary review
Source: Hum Relat. 2025 Dec 30;79(2):246–78. doi: 10.1177/00187267251403902 (PMC12812018; doi:10.1177/00187267251403902)
Supplement: sj-docx-2-hum-10.1177_00187267251403902 – Supplemental material for Problematizing the role of artificial intelligence in hiring and organizational inequalities: A multidisciplinary review [file sj-docx-2-hum-10.1177_00187267251403902.docx]

**Supplemental material B: Summary of the reviewed articles in the final corpus.**

| Publication | Cluster | Key foci | Key concepts ^1^ |
| --- | --- | --- | --- |
| Acikgoz et al. (2020) | HRMOS | Perceptions of AI | AI, ‘AI life cycle’, fairness, justice, perceptions, trust, selection decisions |
| Adams-Prassl (2019) | LS | Algorithmic management | Algorithmic management, control, data protection, discrimination, fairness, Polyani’s paradox, privacy, responsibility |
| Aizenberg and Van Den Hoven (2020) | SS | Human rights implications of AI | AI, accountability, fairness, human rights, privacy |
| Ajunwa (2020a) | LS | Implications of algorithmic hiring for inequality and discrimination | Algorithmic hiring, anti-bias, automation, bias, cultural fit, data, disparate treatment and disparate impact, discrimination, efficiency, fairness, law, platform authoritarianism |
| Ajunwa (2020b) | SS | Implications of algorithmic hiring for inequality and discrimination | Algorithmic hiring, algorithmic invisibility, black box, ‘data laundering’, discrimination, surveillance |
| Ajunwa (2021a) | LS | Implications of algorithmic hiring for inequality and discrimination | AI, algorithmic hiring, bias, discrimination, fairness, legal accountability, regulation, technical transparency |
| Ajunwa (2021b) | LS | Ethical and legal implications of automated video interviewing (AVI) | Anti-discrimination law, automated hiring, automated video interviewing (AVI), bias, discrimination, fairness, hiring, privacy, regulation |
| Ajunwa and Greene (2019) | SS | Automated hiring platforms | Automated hiring platforms, digital intermediaries, platform authoritarianism, management, work platforms |
| Allal-Chérif et al. (2021) | HRMOS | Digital technologies and recruitment | AI, automation, decision-making process, digital technologies, e-recruitment, efficiency, human bias, human oversight |
| Amershi et al. (2014) | CS | Roles of humans in  interactive machine learning | Humans, intelligent systems, interactive machine learning, systems, users |
| Andrews and Bucher (2022) | LS | Implications of algorithmic hiring for inequality and discrimination | AI, algorithm training, automated resume screening, video interviewing, video games, bias, data, discrimination, gender inequality, hiring |
| Baer (2019) | CS | Measuring and mitigating algorithmic bias | AI, algorithmic bias detection, debiasing, mitigation, prevention |
| Bankins (2021) | HRMOS | Ethical use of AI in human resource management (HRM) | AI, accountability, bias, decision-making process, fairness, ethical AI, HRM, task-technology fit |
| Bankins et al. (2024) | HRMOS | Algorithmic management | AI, algorithmic management, ethics, employee interactions, human-AI collaboration, perceptions, platform‐based work, work |
| Basu et al. (2023) | HRMOS | Implications of AI for HRM | AI, AI-HRM interaction, bias, efficiency, employee engagement, HRM, human oversight, governing, organizational performance |
| Belenguer (2022) | CS | Machine-centric solutions to algorithmic bias | AI, algorithmic bias, decision-making, fairness, human-centric solutions, machine-centric solutions, testing, transparency, validation |
| Benbaya et al. (2020) | HRMOS | Implications of AI in organizational decision-making | AI, data, decision-making, explainability, ethics, organizations, transparency |
| Bent (2020) | LS | Implications of algorithmic affirmative action | Algorithmic affirmative action, anti-discrimination law, bias, fairness, governmental actors/interest/use, discrimination |
| Birhane and Cummins (2019) | CS | Algorithmic bias | Algorithmic decision-making, injustice, automated systems, bias, efficiency, relational ethics/justice, technical solutions |
| Black and van Esch (2020) | HRMOS | Implications of AI for hiring | AI-enabled recruiting, bias, data-driven insights, decision-making, digital recruiting technology, efficiency, human resources |
| Blass (2019) | LS | Algorithmic advertising discrimination | Algorithmic accountability, algorithmic advertising, data, discrimination, legal implications, social media, transparency |
| Bloch-Wehba (2022) | LS | Algorithmic governance and regulation | Algorithmic governance, algorithmic audits, bias, discrimination, oversight, regulation |
| Borges et al (2021) | HRMOS | Implications of AI in organizational decision-making | AI, advantages, automation, business strategy, decision-making |
| Bornstein (2018) | LS | Algorithmic discrimination | AI, algorithmic discrimination, anti-discriminatory algorithms, anti-stereotyping, bias, decision-making processes, disparate treatment, regulation |
| Budhwar et al. (2022) | HRMOS | Implications of AI in transforming HRM practices | AI, AI-based applications, data privacy, decision-making processes, ethical, legal and moral concerns, global context, HRM, talent management |
| Chang and Ke (2024) | HRMOS | Implications of AI in transforming HRM practices | AI, equality, ethics, fairness, human resource management (HRM), inclusivity, organizations, people analytics (PA), socially responsible AI (SRAI) |
| Chen (2023a) | CS | AI solutions for mitigating human bias in recruitment | AI, employment, human bias, human judgment, human prejudice, recruitment, talent acquisition |
| Chen (2023b) | SS | Implications of algorithmic hiring for inequality and discrimination | AI-enabled recruitment, algorithmic bias and discrimination, algorithm designers, data, ethical governance, external oversight, perceptions |
| Cofone (2018) | LS | Algorithmic discrimination with a focus on data | Algorithmic discrimination, anti-discrimination law, data governance, disparate treatment, fairness, information, privacy, regulation, transparency |
| Crawford et al. (2019) | SS | Social and ethical implications of AI technologies | Algorithmic accountability, AI, bias, corporate ethics, discrimination, ethics, law, policy, power, transparency |
| Cruz (2024) | SS | Role of experts in defining concepts of fairness in algorithmic hiring | AI-based hiring decisions, experts, fairness, hiring, organizations |
| Dattner et al. (2019) | HRMOS | Legal and ethical implications of using AI in hiring | AI, bias, data, decision-making, ethical and legal implications, hiring, privacy |
| de Alford et al. (2020) | CS | Algorithmic bias in machine learning | AI, adversarial learning, age bias, demographic parity, ethics, fairness, machine learning, model accuracy, prediction |
| De Cremer and De Schutter (2021) | CS | Algorithmic decision-making | Algorithmic decision-making, data, diversity, human bias, inclusiveness, organizations, recruitment |
| Drage and Mackereth (2022) | SS | AI implications for hiring/recruitment bias | AI, bias, debiasing, discrimination, eradication of difference, gender, HR, inequality, race, recruitment |
| Dries et al. (2023) | HRMOS | AI and the future of work | AI, automation, future of work, job, robot, technology, transformation, workplace |
| Dwivedi et al. (2019) | HRMOS | Implications of AI for organizations, industry, society | AI, accountability, augmentation, autonomous intelligence systems, bias, business, ethics, governance, implications, machine-learning, privacy, productivity, replacing human tasks, regulation, responsibility, safety |
| Einola and Khoreva (2023) | HRMOS | Human-AI collaboration in HRM | AI, augmentation, automation, human-AI collaboration/co-existence, HRM, organizations |
| Elliott (2019) | SS | Implications of AI for culture and society | AI, automation, big data, culture, digital era, employment, humans, machines, social interactions, self and private life, surveillance, work |
| Favaretto et al. (2019) | CS | Implications of big data for inequalities | Algorithmic processing, bias, big data, data mining, discrimination, disparity, fairness, inequality |
| Fernández-Macías et al. (2018) | CS | AI and the future of work | AI, alteration, automation, autonomy, generality, occupations, organizations, skills, socio-economic and computational perspectives, task |
| Friedman and McCarthy (2020) | LS | Implications of AI for employment law and regulation | AI, audits, bias, discrimination, employment law, hiring, machine learning, regulation |
| Fritts and Cabrera (2021) | SS | Implications of AI for dehumanisation in hiring | AI, dehumanisation, employee-employer relationship, ethics, evaluation, human judgment, recruitment, screening |
| Fuchs (2023) | LS | Implications of AI for employment law and regulation | AI, anti-discrimination laws, bias, discrimination, fairness, hiring, legal accountability, New York City law, regulation |
| Galerpin (2019) | SS | Implications of online hiring platforms for gender discrimination | Gender segregation, gender stereotypes/discrimination, gig economy, online hiring |
| Gelles et al. (2018) | SS | Perceptions of AI | AI, Applicant Tracking Systems (ATS), complexity, fairness, hiring, perceptions, transparency, trust |
| Geyik et al. (2019) | CS | Measuring and mitigating algorithmic bias | AI, debiasing, demographic parity, equality of opportunity, fairness-aware ranking, quantification and measurement of bias, LinkedIn talent search, protected attributes |
| Glymour and Herington (2019) | CS | Measuring and mitigating algorithmic bias | AI, algorithmic bias, bias mitigation, behavior-relative error bias, disparate impact, disparate treatment, procedural bias, outcome bias, score-relative error bias |
| Gonen and Goldberg (2019) | CS | Measuring and mitigating bias in word embeddings | Debiasing, gender biases, natural language processing (NLP), text corpora, word embeddings |
| Hacker (2018) | LS | Implications of AI for employment law and regulation | AI, algorithmic audits, algorithmic decision-making, algorithmic discrimination, algorithmic fairness, data protection law, GDPR, EU anti-discrimination law, regulation |
| Hensler (2019) | LS | Implications of AI for employment law and regulation | AI, algorithmic discrimination, anti-discrimination law, auditing, data, discrimination, equality, GDPR, proactive regulation |
| Huang and Rust (2018) | HRMOS | Human-AI collaboration | AI, human-AI collaboration, innovation, mechanical/analytical/ intuitive and empathetic intelligence, service, task |
| Huang et al. (2019) | HRMOS | Human-AI collaboration | AI intelligences: mechanical, thinking, and feeling, analytical, cognitive, ‘feeling economy’, emotional, empathetic, human workers, interpersonal, tasks |
| Jarrahi (2018) | HRMOS | Human-AI collaboration | AI, decision making, ‘human-AI symbiosis’, human augmentation, machine learning, organizational decision-making, processing capacity |
| Joyce et al. (2021) | SS | Implications of AI for inequality | AI, code, data, inequality, sociology of AI, structural social change |
| Kaminski (2018) | LS | Algorithmic governance | AI, algorithmic accountability, binary governance, decision-making, dignitary, justificatory, and instrumental concerns, GDPR, privacy, regulation |
| Kelan (2023) | HRMOS | Implications of AI for hiring | AI-supported hiring, ‘algorithmic inclusion’, bias, data, design, decisions, diversity, fairness, hiring, inequalities, machine learning, predictive algorithms |
| Kellogg et al. (2020) | HRMOS | Implications of AI for work | AI, algorithmic control, algorithmic occupations, organizational control, power, worker autonomy |
| Kim (2019) | LS | Implications of AI for discrimination and regulation | AI, anti-discrimination law, bias, big data, hiring algorithms, recruitment, protected groups, workplace |
| Kim (2020) | LS | Implications of AI for discrimination and regulation | AI, autonomy, bias, data, equality, fairness, hiring, inequality, labour markets, liability, online manipulation, opportunity markets, predictive algorithms, regulation, transparency |
| Köchling and Wehner (2020) | HRMOS | Algorithmic decision-making | Algorithmic decision-making, discrimination, fairness, HR development, HR recruitment |
| Kordzadeh and Ghasemaghaei (2022) | HRMOS | Implications of algorithmic decision-making for bias and discrimination | Algorithmic accountability, bias, data-driven decision making, discrimination, ethics, fairness, information systems |
| Kuhlman et al. (2020) | CS | Ethical implications of AI | Algorithmic bias, computing, data, diversity, ethics, fairness, representation, structural inequalities, systemic structural biases |
| Langenkamp et al. (2020) | CS | Fairness in algorithmic hiring | AI, algorithmic hiring, automation, fairness, employment decisions, hiring, legal and technological perspectives, machine learning, regulation, transparency |
| Lee et al. (2015) | CS | Algorithmic management | Algorithmic management, data-driven management, human workers, machines, transparency, work practices |
| Lee (2018) | SS | Perceptions of AI | AI, algorithmic management, decision making, emotion, fairness, human empathy, human skills, mechanical skills, perceptions, trust |
| Lepri et al. (2018) | CS | Algorithmic decision-making | Accountability, algorithmic decision-making, fairness, machine learning, technical solutions, transparency |
| Lin et al. (2020) | SS | Implications of AI for implicit bias | AI, engineering equity, human decision-making, human-machine interaction, human resource recruitment, implicit bias |
| Madaio et al. (2022) | CS | Perspectives of AI practitioners | AI systems, ethics, fairness, practitioners, organizational factors |
| Mann and Matzner (2019) | LS | Implications of algorithmic profiling for regulation | Algorithmic profiling, anti-discrimination law, bias, complexity, data protection, discrimination, invisibility, protection, regulation |
| Marti et al. (2024) | HRMOS | AI implementation within organizations | AI, disruptive algorithms, envelopes, fairness, organizational fields, regulation |
| Martin (2019) | HRMOS | Ethical implication of AI | AI accountability, algorithms, bias, decision-making process, ethics, responsibility, transparency |
| Michailidis (2018) | SS | Implications of AI for HR | AI, bias, blockchain, data, employment, HR, recruitment |
| Nachbar (2021) | LS | Algorithmic discrimination and fairness | AI, accountability, algorithmic fairness, computational considerations, decision-making, discrimination, legal considerations, regulation, transparency |
| Nawaz (2019) | HRMOS | Implications of AI for HR | AI, automation, bias, communication, data, decision-making, hiring, human bias, recruitment, screening |
| Newman et al. (2020) | HRMOS | Implications of AI for procedural fairness in hiring processes | Algorithmic decision-making, algorithmic reductionism, bias, fairness, HR decisions, human bias, procedural fairness/justice |
| Osoba et al. (2019) | SS | Implications of AI for equity and fairness | AI, algorithmic equity, algorithmic bias, decision-making, decision pipeline, fairness, hiring, recruitment, social applications, transparency |
| Parviainen (2022) | LS | Implications of AI for employment law and regulation | Algorithmic recruitment, automated decision-making, EU, GDPR, law, regulation |
| Raghavan and Kim (2023) | LS | Algorithmic discrimination and limitation of four-fifths rule | Algorithmic bias, algorithmic hiring, anti-discrimination law, discrimination, fairness, four-fifths rule |
| Raghavan et al. (2020) | CS | Algorithmic bias mitigation | Algorithmic bias, algorithmic hiring, anti-discrimination law, bias, de-biasing, disparate treatment/impact, technical perspectives, training data |
| Raisch and Krakowski (2021) | HRMOS | Human-AI collaboration | AI, automation-augmentation paradox; decision-making, human bias, human involvement, management, tasks, statistical bias |
| Rigotti and Fosch-Villaronga (2024) | LS | Implications of AI for fairness in recruitment | AI, anti-discrimination law, bias, data protection, discrimination, fairness, recruitment, regulation, transparency |
| Robert et al. (2020) | SS | Implications of AI for fairness in management | AI, autonomy, bias, decision-making, fairness, organizations, trust |
| Roemmich et al. (2023) | CS | AI as a technical solution for organizational problems | AI, bias, ‘emotion artificial intelligence’, hiring, organizational problems, technosolutions |
| Rosenblat et al. (2014) | SS | Implications of data-driven recruitment for discrimination | Algorithms, Applicant Tracking Systems (ATS), bias, data-driven recruitment, data, ‘networked’ employment discrimination |
| Rovatsos et al. (2019) | SS | Implications of algorithmic decision-making for bias and discrimination | Algorithmic bias, bias mitigation, data protection, decision-making, discrimination, fairness, GDPR |
| Shrestha et al. (2019) | HRMOS | Human-AI collaboration | AI, human-AI collaboration, organizational decision-making |
| Sonderling et al. (2022) | LS | Implications of AI for employment law and regulation | AI, accountability, anti-discrimination law, bias, employment discrimination, fairness, regulation, transparency |
| Tambe et al. (2019) | HRMOS | Implications of AI for HR | AI, bias, decision-making, discrimination, HR, performance evaluation, talent acquisition |
| Todoli-Signes (2019) | SS | Implications of AI for reinforcing bias/discrimination | AI, algorithms, automated decision-making, data protection, discrimination, GDPR, governance, regulation |
| Upadhyay and Khandelwal (2018) | HRMOS | Implications of AI for hiring | AI, automation, bias, hiring, recruitment, repetitive tasks |
| Wajcman (2017) | SS | Social and economic implications of technological change | AI, automation, future of work, politics of technology, power, robotics |
| Weiskopf and Hansen (2023) | HRMOS | Algorithmic governmentality | Accountability, algorithmic governmentality, bias, decision-making, ethics, people analytics, transparency |
| Xiang (2021) | LS | Legal and technical approaches to algorithmic bias | AI, algorithmic bias, bias mitigation, anti-discrimination law, data, fairness, legal perspective, liability, technical perspective |
| Yu et al. (2018) | CS | Ethical implications of AI | AI, accountability, bias, ethics |
| Yu (2020) | LS | Implications of AI for inequality | AI, accountability, ‘algorithmic divide’, bias, data, ‘digital divide’, equality, governance, regulation |
| Zuboff (2019) | SS | Social and economic implications of technological change | Behavioural traces, data extraction, digitization, power, privacy, surveillance |
| ^1^ The concepts that appear in quotation marks are original terms used by the authors | | | |
