# Supplementary material for: Problematizing the role of artificial intelligence in hiring and organizational inequalities: A multidisciplinary review
Source: Hum Relat. 2025 Dec 30;79(2):246–78. doi: 10.1177/00187267251403902 (PMC12812018; doi:10.1177/00187267251403902)
Supplement: sj-docx-3-hum-10.1177_00187267251403902 – Supplemental material for Problematizing the role of artificial intelligence in hiring and organizational inequalities: A multidisciplinary review [file sj-docx-3-hum-10.1177_00187267251403902.docx]

**Supplemental Materials C: Further Information on the Two-Phase Review Process**

**Introduction**

In this supplemental material, we elaborate on the methods and decisions underpinning our two-phase review process: a scoping review (Phase 1) and a problematizing review (Phase 2). This hybrid methodology combines the strengths of both approaches. The scoping review allowed us to establish our multidisciplinary review corpus in a systematic fashion, capturing the breadth of the emergent scholarship on AI, hiring and inequality. The problematizing approach enabled us to go beyond mere description and engage more deeply with scholarship, to establish a critical multidisciplinary dialogue.

Our hybrid approach is particularly valuable because of the complexity of scholarship on AI, hiring and inequality (e.g., rapidly emerging, dynamic, multidisciplinary, multimethod). In the sections below, we offer a step-by-step justification and elaboration of our review approach. We begin by addressing the disciplinary positionality of our four-author team and our epistemological approach. We then provide details on the two phases of our hybrid approach.

**Positionality**

This review was conducted by a team of social scientists with overlapping expertise and shared interests in social, organizational and labour market inequalities. Our perspective has been shaped by prior collaboration with scholars from other disciplines (e.g., computing, statistics, management, information systems) in a project examining the implications of AI for labour market (in)equality. The specific impetus for a multidisciplinary review emerged as a result of our experiences working on this larger project, including challenges encountered and solutions developed, to establish a common, situated, and systematic understanding of the role of AI in (re)producing labour market inequalities.

We follow the ideas of Alvesson and Sandberg (2020) that the reviewer is never neutral in guiding the review (p. 1295) and that reflexivity is central to knowledge production (p. 1297). Here we discuss how our positionality as social scientists shaped the way we interacted, analyzed and understood the scholarship emerging across various disciplines on the role of AI in shaping/reshaping hiring processes and inequality embedded in these processes.

Our engagement with the topic of AI, hiring, and inequality is shaped by a critical lens that incorporates reflexivity and attention to structure. We view inequality as a structural, pervasive societal issue (e.g., Acker’s inequality regimes) and focus on the role of technology in interacting with and reproducing it. From this perspective, AI systems are not neutral tools, but are shaped by—and help to reproduce—historical inequalities tied to gender, race, and class, gender. For example, we reflect on how the notion of ‘algorithmic bias’ is often framed in narrow or technical terms, without considering the deeper, pervasive social structure that underlies it.

As discussed in the paper, our analytical process in Phase 2 of the review (the problematizing phase) involved developing and accumulating expertise within each disciplinary cluster by assigning and maintaining a ‘primary reader’ role, where the reader immerses themselves in the particular disciplinary cluster (e.g., CS, HRMOS, SS, and LS). Additionally, each author rotated across other disciplinary clusters as a ‘secondary reader’ to facilitate an interdisciplinary understanding of this scholarship.

While each of us has developed expertise on the CS, HRMOS, SS or LS scholarship for the duration of our review and as a part of our bigger project, we also acknowledge that our original expertise, training and experience have shaped our evolving individual and collective understanding of the intra- and inter-disciplinary insights concerning AI, hiring and inequality. For example, our positionality is reflected in our interest in and focus on hiring as a foundational asymmetric process within market-based, capitalist economies that structures access to opportunities and has profound implications for the lives of individuals, their well-being, and economic security. Our choice of the key texts for problematizing reflects our awareness of, and interest in, the pervasiveness of organizational inequality (Acker, 2006), within capitalist structures marked by power asymmetries (Zuboff, 2019; Pasquale, 2015). Finally, our choice of hybrid methodology that combines scoping and problematizing methods highlights our interest in *how* knowledge is being created, in addition to *what* knowledge is being created (Alvesson and Sköldberg, 2018)

**Epistemology**

Our epistemological stance draws on ideas of reflexive methodology (Alvesson and Sköldberg, 2018), which emphasizes the importance of moving between empirical analysis, theoretical interpretation, and reflection on our own role as researchers. Following our disciplinary positionality as scholars of social, organizational, and labour market inequality, our review is inspired by critical and constructivist understandings of knowledge production. We recognize that knowledge about AI, hiring, and inequality is not simply discovered but is actively constructed through debates, across disciplines, domains and stakeholders, and that different approaches and assumptions guide these debates. Thus, our review is not a neutral summary or description of a found field of knowledge, but is a situated contribution embedded in our disciplinary backgrounds and research decisions. Consequently, our choices—what we include, what we leave out, and how we analyze data—are shaped by our standpoints, as scholars and individuals. As social scientists, we approach AI, hiring, and inequality not as isolated technical phenomena, but as part of broader organizational, social and political structures. As individuals residing in the Global North, with diverse transnational origins, educational training, and experiences, we are aware of how our perspectives are shaped by the debates and discourses that prevail in this part of the world. Our status as academics facilitates participation in these debates.

Alvesson and Sandberg (2020) underscore the reviewer’s role “as an artist, a detective, an innovator or even an anthropologist, supporting the innovative part of research” (1302). In line with their problematizing methodology, we go beyond ‘representing’ a field of scholarship. Using a hybrid approach we aim to utilize scoping techniques to map the emerging knowledge and its construction, and to utilize problematizing techniques to critically engage with the assumptions that underlie how AI and inequality are currently studied in the context of hiring and organizations. Our goal is thus to ‘open up’ space for new questions and to shift how key issues are being approached in this field.

**Overview of our hybrid process**

**Phase 1: Scoping review:**

The goal of this phase was to identify the breadth of emerging ideas and debates on the intersecting domains broadly defined as AI, hiring practices and inequality (AI / hiring / inequality). A scoping methodology was chosen as the most suitable method for assembling literature and mapping key ideas in rapidly emerging fields, which are not stable and complex (Mays et al., 2001:194). The knowledge emerging at the intersection of AI, hiring practices, and inequality is such a field, and a scoping approach was essential to assess the breadth of the field to paint a representative picture of the emerging knowledge. The scoping approach combines rigour with flexibility, allowing for the collection of emerging work across disciplines and methods (Arksey and O’Malley, 2005; Daudt et al., 2013). To maintain rigour in the process of selecting and mapping the scholarship across disciplines and methods, we adapted the 5-stage framework of Arksey and O’Malley (2005), who contributed to the methodology of the scoping review and identified ways in which the scoping method is different and similar to the systematic review process.

***Step 1: Formulating research questions*:** To begin, we formulated several broad questions that our review seeks to answer: What disciplines are involved in studying the role of AI in hiring and recruitment? What questions are being asked? What is known about the capacity of AI to reproduce, mitigate or form new forms of inequality? These questions guide our subsequent literature search and review strategies. Following scoping methodology, our questions were broad enough to capture the breadth of the emerging ideas and debates.

***Step 2: Search:*** To operationalize our questions, we used the combination of keywords from the following areas: 1) *algorithms* and *AI*; 2) *human resource management*, *hiring*, *recruitment*, *work, employment* and *organizational* processes; and 3) *(in)equality, fairness, bias, stereotypes, prejudice* and *discrimination* (in short, an intersection of 3 domains: AI/hiring/inequality) to search for literature on Google Scholar, followed by ProQuest, Sociological Abstracts, Web of Science Social Sciences Citation Index, Sage Journals, and Social Sciences Research Network.

We used broad criteria to identify distinct types of publications, questions, and ideas, and we did not incorporate filters of ‘quality’ that are typical for systematic reviews (e.g., limiting the corpus to certain databases). This approach is particularly suitable for our multidisciplinary review due to different publication traditions across different disciplines. For example, CS papers are typically published as peer-reviewed conference proceedings indexed by Google Scholar but not by conventional databases such as Scopus and the Web of Science. In this case, a database-informed approach, though widely used in previous single-discipline reviews, would not be suitable for establishing a quality filter for our corpus. Rather, we more specifically ensured that the individual entries included in our review corpus are of high quality— e.g., they are published in widely recognized and credible outlets (e.g., journals, conference proceedings). We screened titles and abstracts to identify the substantive relevance of the items to the subject matter. This search resulted in 389 publications.

***Step 3: Selection and screening:*** The 389 publications were exported to the Rayyan platform to allow for blind inter-rater screening. Our authorship team then screened these 389 publications, using individual blind selection. Each author screened the 389 publications and assigned the label of either ‘included’ (criteria: highly relevant to our review), ‘excluded’ (criteria: not very relevant to our review) or ‘maybe’ (criteria: somewhat relevant to our review). The notion of ‘relevance’ was defined as substantive relevance to the intersection of 3 domains of interest (AI/hiring/inequality). The ‘relevance’ was defined as a substantive engagement with the 3 domains of interest. For example, publications that discussed AI and inequality in the context of hiring/workplaces/organizations were tagged as ‘highly relevant’.

Inclusion criteria: inter-rater agreement on the highest relevance to the intersection of the domains, broadly defined as AI/hiring/inequality.

Exclusion criteria: inter-rater agreement on low-medium relevance to the intersection of the domains, broadly defined as AI/hiring/inequality.

The final decision on a label was based on the majority coding. In the case of a tie between labels, we adopted an inclusive approach to ensure that we did not miss out on important publications. For example, if the tie was between ‘excluded’ and ‘maybe’, the final decision would be ‘maybe’. If the split was between ‘maybe’ and ‘included’, the final decision was ‘included’.

Of the items reviewed, 75% reached a majority agreement (3/4 reviewers) reflecting strong inter-rater reliability in the selection process. We proceeded with the category of included, which consisted of 212 empirical, conceptual, and applied publications in different disciplines.

***Step 4: Charting the data*:** We then created a detailed mapping of the 212 publications, noting discipline, key questions, concepts and insights. Based on this mapping, we identified four disciplinary clusters connected to 1) computing and data sciences (CS); 2) human resource, management and organization studies (HRMOS); 3) other social sciences (SS); and 4) legal scholarship (LS). As noted in our paper, we do not view the borders of disciplinary clusters as rigid, given the complexity and novelty of emerging knowledge and given the emerging communication across disciplines.

The cluster that we identified as **Computing and Data Sciences (CS**) is limited to literature that incorporates social aspects/concerns/perspectives along with technical perspectives, thus excluding a vast computer science literature that engages only with technical perspectives. The computer science discipline currently dominates knowledge generation on AI, focusing predominantly on the technical aspects of algorithms. Therefore, it is important to note that our review engages with the slice of this scholarship that speaks to social perspectives along with technosolutions.

The cluster that we identify as **Human Resource, Management, and Organization Studies (HRMOS)** contains both applied and critical writings that centre on human resource practices in organizations, with a special focus on AI applications and implementation in HR processes. We identify this scholarship as a unique cluster and single it out from the broader social sciences (SS) debates.

The cluster we identify as **Social Sciences (SS)** is a heterogeneous cluster that combines discussions and debates from various fields of sociology, psychology, philosophy, and science and technology studies (STS). While coming from diverse disciplines, these debates offer a broader, critical lens on matters of AI and the social implications of its usage.

The cluster we identify as **Legal Scholarship (LS)** offers very focused writing on legal issues, centring on Western, North American, and European perspectives. The regional foci of this body of literature are partly a result of our focus on publications in the English language only, which we have acknowledged as a limitation of our review in the main article.

In a small number of cases where an item could potentially belong to more than one cluster (i.e., socio-technical perspectives which draw expertise from the CS and SS), we assigned it to the cluster that was most substantively relevant, such as the key focus of the paper, the scholarship that the paper engages with and the expertise of the authors.

The distribution of the 212 items by discipline is as follows:

| **Discipline** | **Number** | **Percentage** |
| --- | --- | --- |
| CS | 47 | 22.2% |
| HRMOS | 73 | 34.4% |
| SS | 55 | 25.9% |
| LS | 37 | 17.5% |
| Total | 212 | 100.0% |

**Phase 2: Problematizing review**

Phase 1 highlighted that the literature emerging on the intersection of AI/hiring/inequality is multidisciplinary and multi-method, and it touches on the phenomena (AI/hiring/inequality) from various angles, fragmented and therefore very complex. The goal of Phase 2 was to gain a deeper understanding of these debates and to critically interrogate the underlying assumptions that drive questions and concerns across the disciplinary clusters we have identified. The goal was to engage with the scholarship more analytically, selectively, and critically, problematizing *what* is known and *how* it is known, with the attempt to identify novel ways of understanding and conceptualizing the role of AI in hiring and inequality.

In this phase, we followed a problematizing review, developed by Alvesson and Sandberg (2020), which was chosen as the most suitable methodology for this phase, as it provides a methodological and epistemological framework for the deep, reflexive analysis of the scholarship. This type of analysis goes beyond a scoping review, which focuses on breadth rather than depth. As Alvesson and Sandberg (2011: 32) clarify, the problematizing is not ‘an end in itself’ but rather a ‘means to identify and challenge assumptions underlying existing theory and, based on that, being able to formulate more informed and novel research questions’.

In this phase, we followed four core principles of problematizing review: 1) ‘reflexive reading’; 2) ‘less is more’; 3) ‘reading broadly but selectively’; and 4) ‘not accumulating but problematizing’ (Alvesson and Sandberg, 2020, p. 1297).

***Principle 1:*** Alvesson and Sandberg (2020) suggest that ‘reflexivity typically calls for the researcher to read a limited number of texts carefully, to challenge his or her interpretations by considering alternative perspectives and sources of inspiration, to work with doubt and recognize intuition, and to aim for insightfulness rather than rigour or pseudo-rigour’ (p. 1297). Recognizing the importance of reflexivity, in Phase 2, we relied more heavily on the knowledge acquired in the first stage of the review and, deep, reflexive and layered reading in the second phase of the review.

We practised reflexivity by assigning a ‘primary reader’ to each disciplinary cluster. The ‘primary reader’ immersed themselves in the disciplinary cluster to develop and maintain the intra-disciplinary expertise’ throughout the duration of the selection and analysis conducted in phase 2. Additionally, we assigned ‘secondary readers’ who rotated across disciplinary clusters, providing further insight. This exercise allowed us to generate and accumulate insight within and across the disciplinary clusters and identify connections between them, the similarities and differences of the ideas, the underlying assumptions that shape these ideas, and the complex ways in which these ideas and assumptions interact. As in the case of reflexive, critical, qualitative data analysis and coding, the primary and secondary readers first assessed their assigned publications separately. They then engaged in deliberation to finalize their analysis of the supercorpus. This deliberation, sustained through numerous written and oral discussions, allowed the reviewers to reflect on their disciplinary/expertise positionality, perspectives and subjectivity. For example, our finding on the asymmetry in knowledge generation and the domination of the computer science perspective was only possible to be identified via multiple layered readings and interpretations of knowledge generated inside and across clusters.

***Principle 2***: Following the idea of ‘less is more’, we created a ‘supercorpus’ – a selection of readings from the scoping review stage (57 items) and additional readings from reading beyond the corpus (40 items).

The 57 items selected from the original corpus represent key ideas and debates emerging from each discipline. The key criteria for inclusion/exclusion in Phase 2 were identifying the most insightful contribution, such as representing a key debate, initiating a new concept/debate, and featuring high substantive relevance to the intersection of AI/hiring/inequality.

As a practice, this selection was operationalized in accordance with the characteristics of the cluster itself. We relied on the accumulated expertise of the ‘primary reader’– the reviewer who immersed themselves in the original disciplinary cluster and gained a broad understanding of the key questions and debates. Each ‘primary reader’ suggested a list of 10-15 articles from their disciplinary cluster for deeper examination. It is important to note, that, epistemologically, our focus in this phase shifted towards a deep, reflexive, layered reading of the small collection of items. In such a process, as previously mentioned, the reviewer is not seen as a neutral ‘puzzle solver’ but rather a ‘creative artist’ whose previous and emerging expertise and positionality shape the way they interact with the scholarship. To enhance rigour, each reviewer was rotating across other disciplinary clusters as a ‘secondary reader’, to meaningfully engage in interdisciplinary analysis.

The disciplinary breakdown of these 57 items is:

| **Discipline** | **Number** | **Percentage** |
| --- | --- | --- |
| CS | 12 | 21.1% |
| HRMOS | 14 | 24.6% |
| SS | 15 | 26.3% |
| LS | 16 | 28.1% |
| Total | 57 | 100.0% |

***Principle 3:*** Following the idea of reading broadly but selectively, we also read beyond the initial corpus and searched for additional scholarship in each discipline, with the aim of expanding and updating the identified debates.

This strategy was adopted for several reasons: (1) AI and inequality in labour market processes is a rapidly evolving field and, as we worked on our review paper, new important publications emerged; (2) ongoing research on AI also relates to classic literature that is not directly related to AI but concerns technological change and its implications for organizational inequalities; and (3) our core selection was strongly tied to the intersection of three domains (AI/hiring/inequality), while other items, in neighbouring or overlapping domains, could be relevant for a deeper understanding of current debates. Our additional search process allowed us to incorporate these key texts and to bring them to bear on the emerging scholarship on AI.

Thus, reading ‘beyond corpus’ was operationalized via: 1) searching the most recent publications related to the intersection of AI/hiring/inequality; and 2) hand searching for items that helped to deepen the discussion on some areas of the intersection, such as broader sociological discussion on implications of AI for society and social inequality that were not directly relevant to hiring. Part of this process involved conducting an additional sweeping search to identify debates that were more conceptual, theoretical and innovative. Similarly, this process of selection was driven by the notion of reflexivity and sustained by the reviewers with accumulated knowledge and expertise across and within disciplinary clusters. As noted previously, epistemologically, the ‘problematizing method’ challenges ideals such as rationality, procedure, transparency, and being trustful of conventions (Alvesson and Sandberg, 2020, p. 14); instead, it draws on the author’s expertise, creativity and reflexivity in the process of selecting and analyzing data. We collected an additional 40 items to be added to our ‘super corpus’ for in-depth examination.

The disciplinary breakdown of these items is as follows:

| **Discipline** | **Number** | **Percentage** |
| --- | --- | --- |
| CS | 7 | 17.5% |
| HRMOS | 18 | 45.0% |
| SS | 5 | 12.5% |
| LS | 10 | 25.0% |
| Total | 40 | 100.0% |

The total number of items forming a ‘supercorpus’ and reviewed in Phase 2 was 97, as reported in the table below, which consists of the selection of readings from the scoping stage and from ‘reading beyond corpus’. The breakdown of the total ‘supercorpus’ is as follows:

| **Discipline** | **Number** | **Percentage** |
| --- | --- | --- |
| CS | 19 | 19.6% |
| HRMOS | 32 | 33.0% |
| SS | 20 | 20.6% |
| LS | 26 | 26.8% |
| Total | 97 | 100.0% |

***Principle 4:*** While in Phase 1, our key goal was to assess the breadth of the accumulating knowledge, in Phase 2, we took an approach of problematizing, which Alvesson and Sandberg (2020) define as an “‘opening up exercise’ that enables researchers to imagine how to rethink existing literature in ways that generate new and ‘better’ ways of thinking about specific phenomena” (page 1291) with the goal of re-evaluating (instead of integrating) existing understandings of phenomena (Alvesson and Sandberg, 2020, p. 1295). This process is identified by analytical and creative rather than systematic evaluation of ideas, and it aims to question and search beneath the more salient ideas.

To facilitate this process, we engaged with theoretical frameworks that help to provide a conceptual lens to understanding the (pre-AI) pervasiveness of inequality in hiring and organizations (Acker, 1990) and the capacity of AI to conceal and reproduce power asymmetries (Zuboff, 2019; Pasquale, 2015). In this step, we used these foundational readings as a prism to gain a deeper understanding, but also ‘develop an alternative assumption ground with the potential to become the start of a novel theoretical contribution’ (Alvesson and Sandberg, 2020, p. 1300) of how AI interacts with inequality regimes.

A central ambition of problematizing is ‘to generate re-conceptualizations of existing thinking that trigger new ideas and theories’ (Alvesson and Sandberg, 2020, p. 1295). Our review and our engagement with the conceptualizations of Acker (1990), Zuboff (2019), Pasquale (2015) and others allowed us to introduce a new concept – ‘algorithmically-mediated’ inequality regimes to capture the way AI interacts and embeds itself within the inequality regimes conceptualized by Acker (1990) which are now cementing, concealing and legitimizing the inequality mechanisms within the algorithmic complexity and pervasiveness. Our problematizing exercise demonstrates that the ‘algorithmically-mediated’ regimes are identified by increased ‘algorithmic invisibility’ and almost ubiquitous pervasiveness, and, therefore, legitimacy, of algorithmic solutions.

The table (next page) provides a summary of articles by discipline at each phase of the review process.

| **Hybrid Review: Distribution of articles by cluster at each stage of review** | | | | | | |
| --- | --- | --- | --- | --- | --- | --- |
| **PHASE 1** | **Phase 1 Scoping review (articles identified)** | | | | | |
|  |  | **CS** | **HRMOS** | **SS** | **LS** | **Total** |
|  | Number | 47 | 73 | 55 | 37 | 212 |
|  | % of Total | 22% | 34% | 26% | 17% | 100% |
|  |  |  |  |  |  |  |
| **PHASE 2** | **Phase 2a Problematizing review (articles selected from initial scoping review above)** | | | | | |
|  |  | **CS** | **HRMOS** | **SS** | **LS** | **Total** |
|  | Number | 12 | 14 | 15 | 16 | 57 |
|  | % of Total | 21% | 25% | 26% | 28% | 100% |
|  |  |  |  |  |  |  |
|  | **Phase 2b Problematizing review (articles identified by reading beyond the corpus)** | | | | | |
|  |  | **CS** | **HRMOS** | **SS** | **LS** | **Total** |
|  | Number | 7 | 18 | 5 | 10 | 40 |
|  | % of Total | 18% | 45% | 13% | 25% | 100% |
|  |  |  |  |  |  |  |
| **FINAL** | **Final Supercorpus (total articles identified)** | | | | | |
|  |  | **CS** | **HRMOS** | **SS** | **LS** | **Total** |
|  | Number | 19 | 32 | 20 | 26 | 97 |
|  | % of Total | 20% | 33% | 21% | 27% | 100% |

**References:**

Acker J (1990) Hierarchies, jobs, bodies: A theory of gendered organizations. *Gender & Society* 4(2): 139-158.

Acker J (2006) Inequality regimes: Gender, class, and race in organizations. *Gender & Society* 20(4): 441-464.

Alvesson M and Sandberg J (2020) The problematizing review: A counterpoint to Elsbach and Van Knippenberg's argument for integrative reviews. *Journal of Management Studies* 57(6): 1290-1304.

Alvesson M and Sköldberg K (2018) *Reflexive Methodology: New Vistas for Qualitative Research*. London: Sage Publications.

Arksey H and O'Malley L (2005) Scoping studies: Towards a methodological framework. *International Journal of Social Research Methodology* 8(1): 19-32.

Daudt HML, van Mossel C and Scott SJ (2013) Enhancing the scoping study methodology: A large, inter-professional team's experience with Arksey and O'Malley's framework. *BMC Medical Research Methodology* 13(48): 1-9.

Mays N, Roberts E and Popay J (2001) Synthesising research evidence. In: Allen P, Black N, Clarke A, Fulop N, Anderson S (eds) Studying the organization and delivery of health services. London: Routledge, 188-220.

Pasquale F (2015) The Black Box Society: The Secret Algorithms That Control Money and Information. Cambridge, MA: Harvard University Press.

Sandberg J and Alvesson M (2011) Ways of constructing research questions: Gap-spotting or problematization? *Organization* 18(1): 23-44.

Zuboff S (2019) *The Age of Surveillance Capitalism: The Fight for a Human Future at the New Frontier of Power.* New York, NY: Public Affairs.
