# Supplementary material for: Problematizing the role of artificial intelligence in hiring and organizational inequalities: A multidisciplinary review
Source: Hum Relat. 2025 Dec 30;79(2):246–78. doi: 10.1177/00187267251403902 (PMC12812018; doi:10.1177/00187267251403902)
Supplement: sj-pdf-1-hum-10.1177_00187267251403902 – Supplemental material for Problematizing the role of artificial intelligence in hiring and organizational inequalities: A multidisciplinary review [file sj-pdf-1-hum-10.1177_00187267251403902.pdf]

Supplemental material A: Scoping review – coding scheme, charting the data

Code category	Code name	Description
Bibliography codes	Author	Author names
	Title	Title of publication
	Year	Year of publication
	Journal	Name of journal, conference proceeding or publication venue
	Discipline/cluster	Academic field (e.g., social sciences, computer science)
	Type of manuscript	Type of publication (empirical, review, etc.)
	Methodology	What is the main methodology(ies) employed in the study?
Thematic codes	Key argument	What are the key arguments in this article?
	Key findings	What are the key findings/results?
	Limitations	What are the key limitations/gaps?
	Future research	What directions for future research are identified?
	Additional insights	Is there anything else important about this article?
Conceptual codes	Theoretical framework	What theory/approach is used to frame the study?
	Key concepts	What are the key concepts used/developed in the study?
	Bias terminology	Is the study using the term 'bias' (yes/no)?
	Bias definition	How is bias defined in the article (if applicable)?
	Types of bias	What are the types of bias discussed in the paper (e.g., human bias, implicit bias, etc.)?
	AI terminology	Is the study using the term 'AI', 'algorithm' or else (yes/no)?
	AI definition	How is AI defined in the article (if applicable)?
	Types of AI	Type of AI discussed in the paper (e.g., machine learning, automated hiring, etc.)
	Fairness/ethics terminology	Is the study using the term 'fairness', 'ethics' or else (yes/no)?
	Fairness/ethics definition	How are 'fairness', 'ethics' or else defined in the article (if applicable)?
